# Longitudinal pre-diagnostic blood samples predict osteoporosis risk

**DOI:** 10.1016/j.isci.2026.117737

**Published:** 2026-09-22

**Authors:** Johan K. Lassen, Kirstine L. Nielsen, Lotte Hindhede, Susan Mikkelsen, Bertram Kjerulff, Sisse Rye Ostrowski, Erik Sørensen, Christina Mikkelsen, Ole Birger Vesterager Pedersen, Mie Topholm Bruun, Bitten Aagaard, Mogens Johannsen, Christian Erikstrup, Palle Villesen

**Affiliations:** 1 Bioinformatics Research Center, Aarhus University, Universitetsbyen 81, 3. Building 1872, 8000 Aarhus C, Denmark; 2 Department of Forensic Medicine, Aarhus University, Palle Juul-Jensens Boulevard 99, 8200 Aarhus N, Denmark; 3 Department of Clinical Medicine, Aarhus University, Palle Juul-Jensens Boulevard 82, 8200 Aarhus N, Denmark; 4 Department of Clinical Immunology, Aarhus University Hospital, Aarhus, Denmark; 5 Department of Clinical Immunology, Copenhagen University Hospital, Rigshospitalet, Copenhagen, Denmark; 6 Department of Clinical Medicine, Faculty of Health and Medical Sciences, University of Copenhagen, Copenhagen, Denmark; 7 Department of Clinical Immunology, Zealand University Hospital, Køge, Denmark; 8 Department of Clinical Immunology, Odense University Hospital, Odense, Denmark; 9 Department of Clinical Immunology, Aalborg University Hospital, Aalborg, Denmark; 10 Novo Nordisk Foundation Center for Basic Metabolic Research, Faculty of Health and Medical Science, Copenhagen University, Copenhagen, Denmark

**Keywords:** metabolomics, osteoporosis, degenerative diseases, machine learning, longitudinal sampling, pre-diagnostic sampling, matched controls

## Abstract

Cross-sectional biomarker discovery studies for degenerative diseases are limited by selection bias, post-diagnostic confounding, and high biological variability. Repeated pre-diagnostic sampling can address these limitations. We analyzed 538 plasma metabolomic profiles (1–6 samples per participant, yearly intervals) from 99 pre-diagnostic osteoporosis cases and 99 matched controls in the Danish Blood Donor Study. We compared performance of cross-sectional and longitudinal modeling using least absolute shrinkage and selection operator (LASSO) logistic regression and random forest. Longitudinal LASSO modeling of metabolite trajectories achieved area under the curve (AUC) values between 0.67 and 0.76, outperforming cross-sectional models with AUC values of 0.57–0.63. Batch- and participant-independent validation of the best model demonstrated stable methodology (AUC: 0.70). The longitudinal data revealed that hippuric acid decreased over time in cases while increasing in controls. We conclude that longitudinal sampling costs more per participant, but the substantial information gain makes it a viable strategy when patient recruitment, not sampling capacity, is the bottleneck.

## Highlights

• Longitudinal pre-diagnostic sampling outperformed cross-sectional design (AUC: 0.76 vs. 0.63)
• Hippuric acid rises with age in controls but falls before an osteoporosis diagnosis
• Hold-one-batch-out validation confirmed the model generalizes across analytical batches
• Longitudinal metabolomics suits patient-scarce diseases where recruitment is the bottleneck

## Graphical abstract

**Figure:**
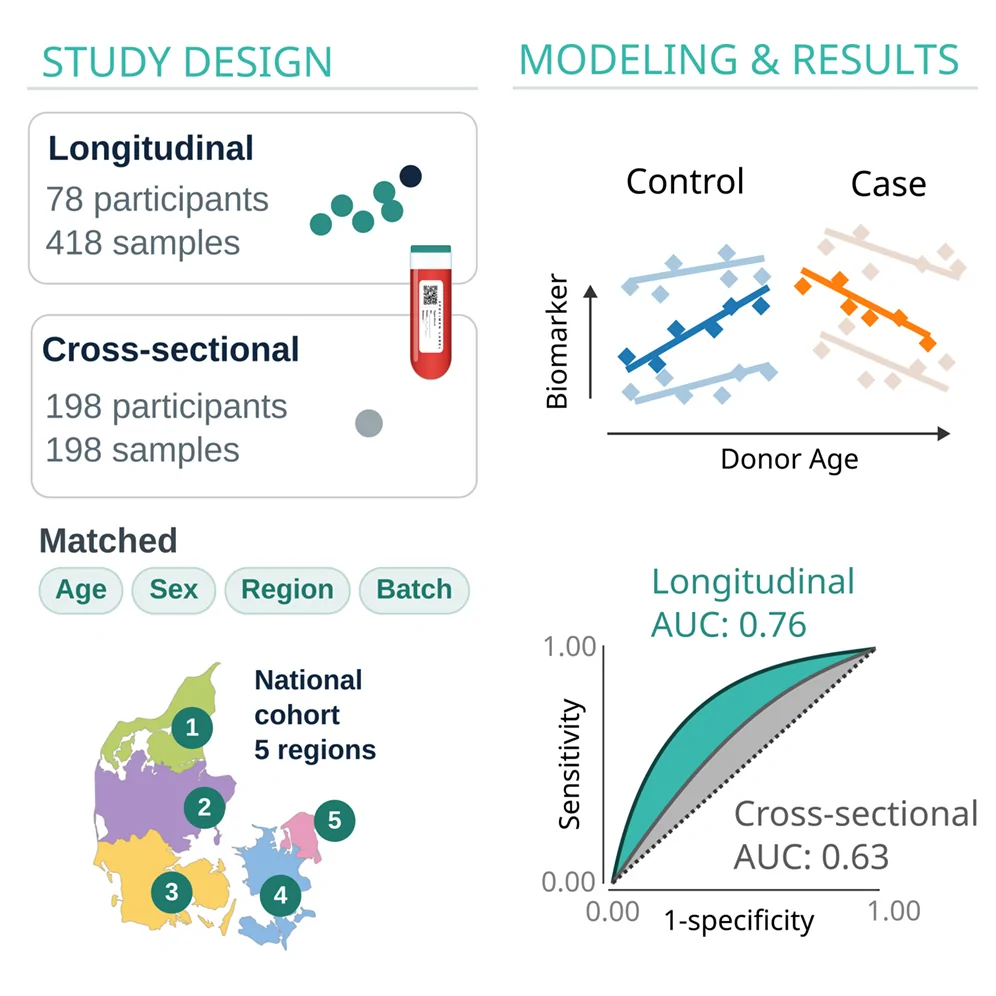

## Teaser

Health sciences

## Introduction

Early identification of individuals at risk for degenerative diseases enables preventive interventions that improve outcomes and reduce healthcare burden. However, finding reliable biomarkers is difficult due to several factors, such as low statistical power, selection bias, and biological variability.^1^

Studies seeking to identify disease biomarkers often use cross-sectional designs, comparing samples from patients and healthy controls at a single time point.^2^ This approach has several limitations, including selection bias caused by differences in traits such as age, lifestyle, medication, or comorbidities. For instance, studies using post-diagnosis samples complicate both the definition of the case group and the construction of suitable control groups. Biomarkers identified in symptomatic patients might reflect non-specific inflammation rather than distinct disease pathology, raising the question of whether control groups should include individuals with other conditions. Day-to-day variability, influenced by factors such as diet, lifestyle, and circadian rhythm, contributes to inter-individual variance and further impedes biomarker discovery.

For osteoporosis, this challenge is particularly relevant as bone mineral density (BMD) loss occurs gradually over decades, tightly associated with metabolic shifts caused by extrinsic and intrinsic factors.^3^ Post-diagnostic samples introduce confounding from fracture events, treatment effects (bisphosphonates and denosumab), and lifestyle changes. Fracture risk assessment tool (FRAX) scores and BMD t-scores require clinical assessment,^4^ limiting population-level screening. Molecular biomarker approaches offer promise for accessible blood-based screening but have shown limited performance gains over conventional risk factors in cross-sectional and observational designs.^5^^,^^6^

We introduce longitudinal and pre-diagnostic samples from the Danish Blood Donor Study (DBDS), focusing on participants later diagnosed with osteoporosis.^7^ The DBDS encompasses over 2.7 million plasma samples from more than 165,000 healthy donors included since 2010 and has previously been used for longitudinal studies.^8^ By linking the DBDS data with national health registries, we identified participants who were diagnosed with osteoporosis after their first donation. We then selected their pre-diagnostic samples and multiple samples from matched controls who did not receive an osteoporosis diagnosis within one year of the last donation. For each participant, we used up to six samples spaced 8 to 14 months apart (Figure 1A) to investigate the potential of longitudinal sampling to find biomarkers using untargeted metabolomics.

**Figure 1:**
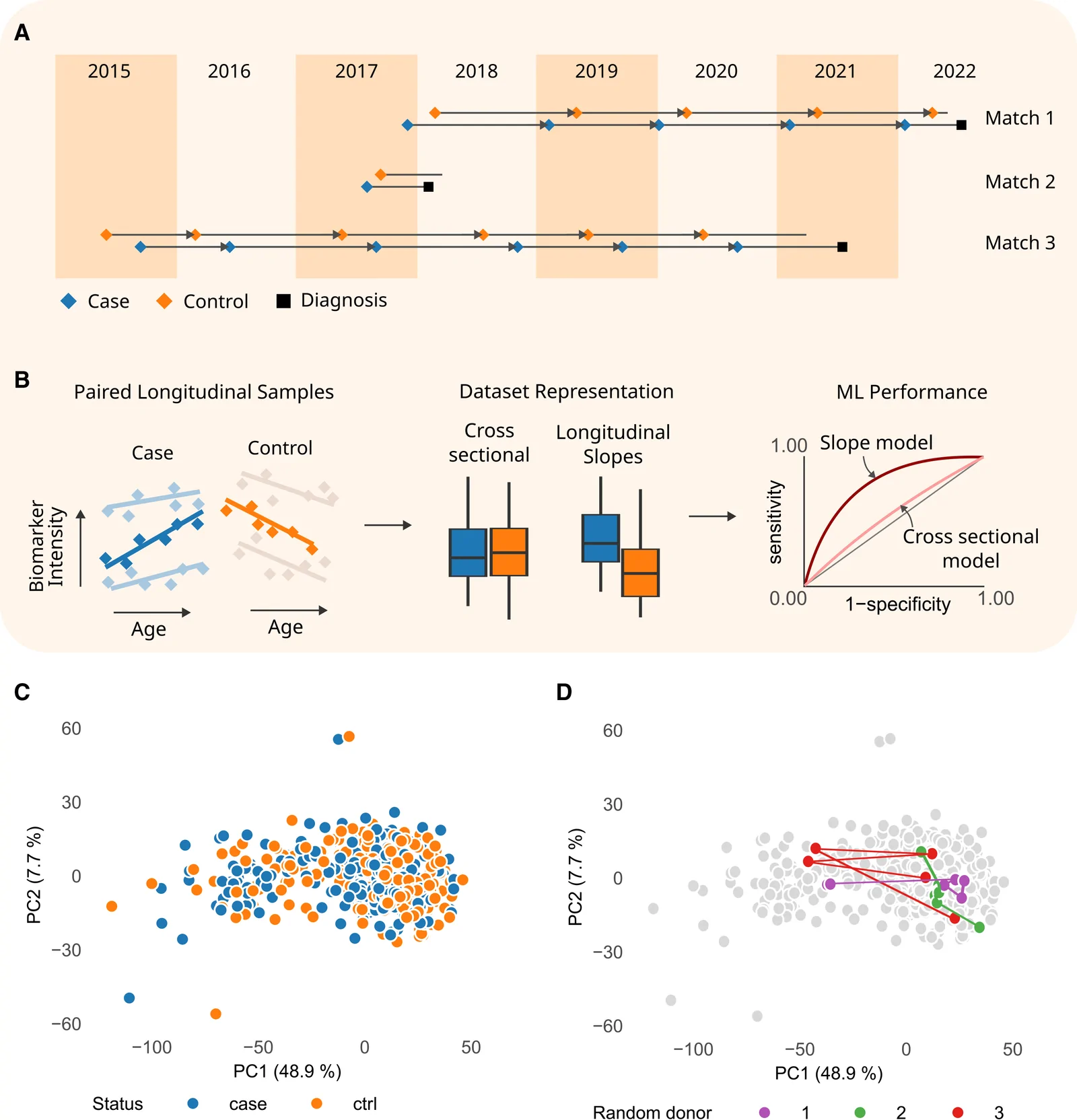
Study design, analytical approaches, and sources of variance (A) Adjacent lines show the donation histories of one matched case-control pair (*n* pairs—match 1: 23, match 2: 60, and match 3: 16). Black squares indicate diagnosis dates. Data points are illustrative (not real). (B) Comparing cross-sectional and longitudinal approaches that use metabolite slopes for machine learning (ML). Illustrative data. (C) PCA of the negative ESI data; single samples colored by case/control. (D) PCA of the negative ESI data with three randomly selected participants highlighted (colored) against all other samples (gray); lines connect each participant’s donations in time. For PCA data, see Data S4.

## Results

Longitudinal modeling outperformed the cross-sectional design, raising performance from an area under the curve (AUC) of 0.63 to 0.76, and identified hippuric acid as the strongest single marker. Next, we present the cohort and metabolome characterization, predictive modeling, robustness testing, and potential biomarkers.

### Unwanted variance hides within and between participant signals

To investigate the potential of longitudinal sampling in untargeted metabolomics, we collected two datasets including 78 participants (39 cases, 39 controls) with longitudinal data and 120 participants (60 cases, 60 controls) with cross-sectional samples (Figure 1). 80% of the cases continued blood donation post-diagnosis, and 96% of the cases were diagnosed without fracture events, indicating early-stage disease in our pre-diagnostic approach.

The analyses captured 3221 features (positive electrospray ionization [ESI]) and 1429 features (negative ESI) after data cleaning and normalization (Figures S1 and S2). Principal component analysis (PCA) showed that cases and controls overlapped in the first six principal components (PCs), with no axis separating the groups (Figures 1C and S3), whereas unwanted variance such as batch effect clustered across PC 1–6 in raw data (Figure S1). Within-participant variance was comparable to between-participant variance as samples from single individuals were as dispersed as samples from the whole dataset. This obscures case-control differences and provides the rationale for longitudinal sampling (Figure 1D). Later components explained the between-individual variance in the negative ESI data (Figure S4).

### Longitudinal study designs outperform cross-sectional designs per participant

We compared two predictive approaches: a cross-sectional approach using the latest time point before diagnosis (*n* = 198 participants) versus a longitudinal approach (*n* = 78 participants, 418 samples in total). The models used only metabolite features, as cases and controls were matched or controlled for sex, age, sampling site, disease history, analytical batch, and sample storage time.

Despite having fewer independent participants, the longitudinal model outperformed the cross-sectional models. The cross-sectional least absolute shrinkage and selection operator (LASSO) models showed near-random performances of AUC 0.63 (95% confidence interval [CI]: 0.55–0.70) and AUC 0.57 (95% CI: 0.49–0.64) for negative and positive ESI data (Figure 2A), indicating minimal metabolite differences at single pre-diagnostic time points. The longitudinal slope-based LASSO models achieved AUC values of 0.76 (95% CI: 0.66–0.87) for negative ESI and 0.67 (95% CI: 0.55–0.79) for positive ESI (Figure 2B; Table S1 for additional metrics). Random forest models performed poorly across all settings (Figure S5), suggesting osteoporosis trajectories are predominantly linear and additive.

**Figure 2:**
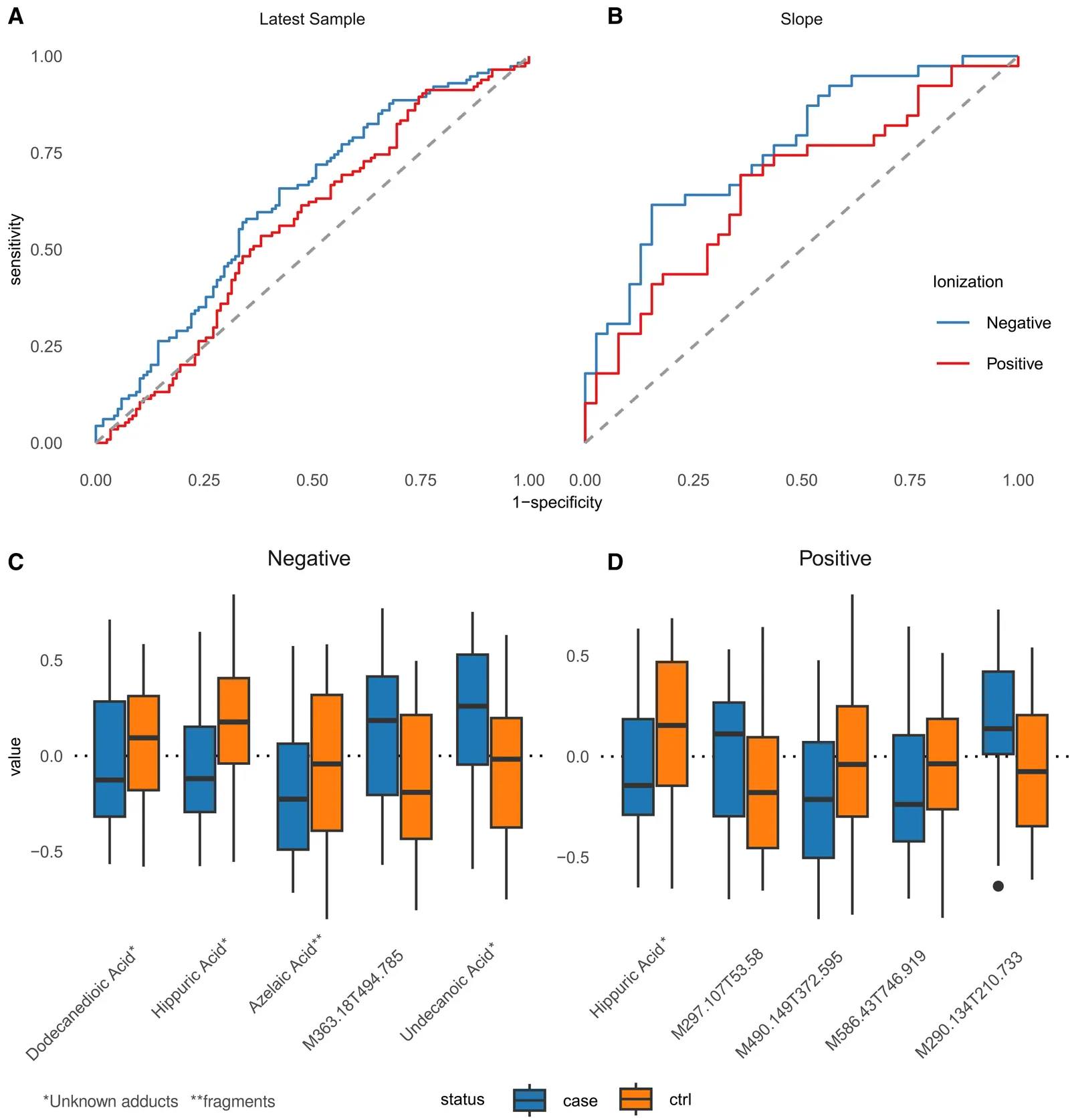
Longitudinal metabolite trajectories predict osteoporosis 1 year pre-diagnosis (A) Leave-one-pair-out cross-validation shows near-random performance for cross-sectional LASSO models. (B) The longitudinal model improves ROC-AUC. (C and D) Top 5 features ranked by median absolute coefficient across cross-validation folds in negative ESI and positive ESI data. See Figure S10 for single-feature ROCs and Data S5 and S6 for ROC values and boxplot data.

Functional data analysis (FDA) models slightly underperformed slopes (AUC 0.70 negative ESI, 0.69 positive ESI), likely because 5–6 time points provide insufficient temporal resolution for functional smoothing. Hence, we find that the simple parametric representations of feature slopes are less prone to overfitting and sufficiently represent rates and directions of the identified features (Figures 2C and 2D). All subsequent analyses focus on slope-based LASSO models.

### Ablation tests of longitudinal study design

To assess how far back in time metabolic trajectories retain predictive signal, we sequentially removed the most recent sample before diagnosis and re-evaluated the model on the negative ESI slope model (Figure S6). The systematic degradation demonstrated detectable metabolic progression 1–2 years pre-diagnosis (200 days AUC = 0.80, 600 days AUC = 0.65), establishing a potential screening window for population-level monitoring. The small increase in AUC between the full data (0.76) and the data upon removal of recent samples (0.80) is likely due to random variance when estimating performance.

We then assessed the performance of the longitudinal model when reducing the sample count to match the number of samples in the cross-sectional model (Figure S6). When removing a matched pair at a time (10–12 samples), we found that the longitudinal model performance reached random chance when having 310 samples available (far above the 198 samples of the cross-sectional data). Here, the data included 18 matched pairs, which did not provide confidence to robustly compare the data budgets between longitudinal and cross-sectional study designs. The reversed test of increasing the 198 cross-sectional samples would have been more suitable, but we did not have these data available. In summary, our results indicate that the longitudinal setting with 78 independent observations extracts more information than the cross-sectional setting with 198 independent observations, meaning more information is extracted per participant in longitudinal metabolomics.

### Generalizability of longitudinal models

Hold-one-batch-out cross-validation yielded AUC 0.70 (*p* = 0.0010, one-sided binomial test, 53/78 successes, null = 0.5) for negative ESI (Figure S7), confirming the model generalizes across independent analytical batches. The batch-blocking design ensures complete independence, as each of the seven batches contains matched case-control pairs never seen by models trained on the other batches. No participant appears in both training and validation sets, eliminating information leakage. The small decrease in performance across batches (AUC 0.70 vs. 0.76) reflects reduced training data. Follow-up studies on the same cohort and analytical platform will likely validate, although we cannot expect generalization to new cohorts.

This robustness has two methodological implications. First, longitudinal designs inherently ignore technical artifacts. Features that fluctuate due to technical confounders will show inconsistent slopes within individuals, which will reduce their model weights. Features that show a more stable and consistent pattern across an individual’s 5–6 donations are more likely biologically meaningful. Second, regression-based data correction successfully normalized batch effects and storage time (STAR Methods, Figures S1 and S2), which dominated the raw variance. Some residual confounding always exists, but the validation demonstrates overall stability.

It should be noted that hold-one-batch-out validation is rare in metabolomics literature. Most studies report within-study cross-validation but avoid batch-wise validation, obscuring technical confounding. Our explicit demonstration of batch robustness addresses generalization issues in metabolomics where promising biomarkers frequently fail external validation.^9^

### Potential biomarkers of early osteoporosis risk

The longitudinal LASSO model selected 25 features in positive ESI mode and 35 features in negative ESI mode, having non-zero coefficients (Data S1). The feature selection sparsity is caused by the L1 penalty inherent to LASSO regression, reducing the risk of overfitting while retaining predictive features. We identified and annotated 25 of the selected features across both datasets (importance in Data S1, annotation in Figure S11, and top 5 in Figures 2C and 2D). Here, several compounds including amino acids (derivatives) and hippuric acid have previously been associated with bone metabolism and osteoporosis.^10^^,^^11^^,^^12^^,^^13^^,^^14^^,^^15^

Hippuric acid had high coefficients in both positive and negative ESI, while demonstrating consistent regulation and effect size, suggesting robustness. We observed a time-dependent increase in the healthy controls and a decrease in the cases (Figures 2C, 2D, and S8). As cases and controls were matched by important confounders such as age, this cannot explain the different slopes of the biomarkers (Figure S9) (as well as for other matching and selection criteria; see STAR Methods).

Analytical selection bias, data filtering, and annotation limitations (typical in untargeted data) caused the absence of known osteoporosis markers such as 25-hydroxyvitamin D, which is essential for calcium absorption,^16^ and estrogen metabolites like 17β-estradiol, known to play a significant role in maintaining bone density.^17^ Markers of bone resorption, including deoxypyridinoline and pyridinoline, which reflect collagen degradation, were also absent.^18^ Despite missing a number of known molecules, we believe the longitudinal model uses biologically relevant features for predicting osteoporosis.

## Discussion

Our longitudinal models capture osteoporosis signal at an early stage as most cases remained healthy enough to continue blood donation post-diagnosis. As osteoporosis affects over 10% of the Danish population and BMD declines progressively with age, some controls likely have undiagnosed early-stage disease.^19^^,^^20^ This creates an inherently challenging classification problem but with potential translational value, as our AUC of 0.76 represents a conservative performance estimate due to potential diagnostic ambiguity.

This observation motivates an interpretational shift from binary classification to acceleration scoring. Just as cholesterol and blood pressure guide cardiovascular disease prevention,^21^ longitudinal metabolic trajectories could track bone health progression. These scores would flag accelerated aging trajectories while individuals remain asymptomatic, enabling early lifestyle interventions to preserve BMD.^22^ Unlike FRAX scores or dual-energy X-ray absorptiometry (DXA) scans,^4^ which are expensive, require clinical visits, and measure outcome rather than progression, metabolic acceleration scores can leverage routine blood samples already collected for other purposes, creating a low-cost screening approach.

Matching on age, sex, sample exclusion, and healthy donor bias produced homogeneous case and control groups, ensuring that age and sex could not drive the model’s predictions and that any discriminative performance reflects the metabolomics data itself. Under the matched design the longitudinal model outperformed the cross-sectional model with fewer independent participants. Hence, cross-sectional metabolomics brings only a marginal improvement to existing covariates, whereas longitudinal data may complement them.

Longitudinal sampling introduces clinical complexity and extra sampling costs, which force a tradeoff between including more participants versus samples per participant; when fixing the longitudinal sample count (18 matched pairs) to the cross-sectional sample count (99 matched pairs) our longitudinal model was random (Figure S8). Hence, longitudinal sampling is most feasible in patient-scarse diseases where recruitment is a bottleneck. These considerations may impede real-world implementation, which should be carefully considered before any deployment.

### Hippuric acid as potential biomarker

Hippuric acid was selected as a predictive feature in both datasets, with a time-dependent increase in the healthy controls, and a decrease in the cases. This corresponds with the literature, which states that hippuric acid increases with age but remains low for individuals with degenerative diseases or frailty.^14^ Chen et al. have published several mechanistic studies indicating that hippuric acid inhibits osteoclast differentiation through GPR109A interactions and further decreases adipogenesis-induced inflammation that also promotes osteoclastogenesis.^15^^,^^23^^,^^24^ Adipogenesis is associated with decreased estrogen during menopause.^24^ Thus, increased hippurate levels reduce bone resorption and support higher BMD.

Hippuric acid is a host-microbe co-metabolite that also associates with visceral fat mass and cardiovascular risk markers.^25^^,^^26^ This broad health impact suggests confounding by lifestyle and we did not carry out mechanistic studies to separate association from causation. Hence, confounding could be caused by pre-diagnostic changes such as diet affecting microbial composition. Longitudinal studies should consider potential confounders in the temporal dimension.

Other studies have also associated amino acid metabolites with osteoporosis.^10^^,^^27^^,^^28^ These studies identify a range of amino acids that affect bone health through, e.g., muscle protein synthesis and collagen structure support, suggesting the association with osteoporosis development, although not agreeing on any specific mechanisms.

### Study design

External validation in outpatient clinics or the general population would test the propesed biomarkers against variance our controlled design excludes, such as high-risk patients and heterogeneity in exercise, diet, and occupation. Appropriate disease controls (orthopedic conditions, other degenerative diseases) would clarify whether the metabolic patterns are specific to osteoporosis or reflect general frailty and aging.

Longitudinal study designs remain uncommon in untargeted metabolomics. One notable study by Ahadi et al. used quarterly sampling over several years to profile aging with untargeted metabolomics.^29^ Other longitudinal studies, including non-metabolomics approaches, employed fewer time points or shorter sampling spans to study, e.g., diabetes-induced polyneuropathy,^30^ Alzheimer’s,^31^ and general health profiles.^32^ Our design of 5–6 time points at yearly intervals represents a practical balance. Future studies should optimize sampling frequency and time span to extend prediction beyond our observed window of 1–2 years and improve robustness. The DBDS has thousands of participants, some having over ten years of donation history, which can address these questions while extending the framework to other degenerative diseases. As populations age, tracking metabolic trajectories in routinely collected blood may shift screening for degenerative disease from diagnosis toward prevention.

### Limitations of the study

Several limitations discussed above are summarized here. Osteoporosis diagnosis relied on registry codes rather than direct BMD measurement, so we could not quantify disease severity. The relatively homogeneous participant population does not reflect the diversity of the general population. Controls were matched on health status rather than including orthopedic or other disease controls, so the model may produce more false positives when applied to a broader population. The study could not establish whether cross-sectional modeling would match longitudinal performance given the same number of samples rather than participants (*n* = 418), as this comparison was not possible with the available data. Analytical selection bias and annotation limitations caused known osteoporosis markers to be absent from the feature set. No mechanistic experiments were performed on hippurate, so association cannot be distinguished from causation. Finally, the cost of repeated sampling and its added complexity may limit real-world implementation.

## Resource availability

### Lead contact

Requests for further information and resources and reagents should be directed to and will be fulfilled by the lead contact, Johan K. Lassen (johan.lassen@forens.au.dk).

### Materials availability

This study did not generate new unique reagents.

### Data and code availability

• Anonymized case-control ratios can be found in Data S2. Note that some rows contain infinite values because of missing matched controls. The DBDS is a platform for studies carried out by the Danish blood centers and collaborators. The study is managed by a steering committee who responds to enquiries regarding collaboration. The blood donors participate in the DBDS to increase the scope of their donation, i.e., to help produce valuable research for the benefit of future patients. Additional information can be found on the homepage (https://www.dbds.dk). DBDS invites researchers to collaborate by contacting the steering committee (info@dbds.dk). Data access requires that projects and applicants obtain permission from the Regional Committees on Health Research Ethics and the Danish Data Protection Agency [https://www.datatilsynet.dk]. Source data for main figures are provided with this paper chronologically in Data S4, S5, and S6.
• All analysis code is provided at Zenodo, https://doi.org/10.5281/zenodo.22269885. Because the underlying feature intensity data cannot be shared, the code is not independently executable throughout. Any additional information required is available from the lead contact upon request.

## Acknowledgments

We thank the blood donors and the staff of the Danish blood centers for making the Danish Blood Donor Study possible. P.V. received funding for J.L.’s PhD from AUFF NOVA (10.13039/501100002739Aarhus University Research Foundation). C.E. has received unrestricted research grants from 10.13039/100014386Abbott Diagnostics and Novo Nordisk (no personal fees).

## Author contributions

C.E., L.H., P.V., K.L.N., and J.K.L. created study design and participant selection. L.H. and J.K.L. coordinated sample identification. K.L.N. and M.J. supervised/acquired measurements. P.V. and J.K.L. performed the statistical analysis and plotting. C.E., P.V., and K.L.N. supervised the data analysis. L.H., S.M., B.K., S.R.O., E.S., C.M., O.B.V.P., M.T.B., B.A., and C.E. organized participant inclusions and data flow in the DBDS. J.K.L. and K.L.N. prepared the initial draft.

## Declaration of interests

C.E. has received research funding from Abbott Diagnostics and Novo Nordisk (no personal fees).

## STAR★Methods

### Key resources table

**Table:** 

REAGENT or RESOURCE	SOURCE	IDENTIFIER
**Biological samples**		

Plasma samples (*n* = 540, 198 participants)	Danish Blood Donor Study (DBDS)	Erikstrup et al.^7^; Ethics: M-20090237, SJ-740, P-2019–99

**Chemicals, peptides, and recombinant proteins**		

Acetonitrile, LC-MS grade	Merck	1.00029
Methanol, LC-MS grade	Merck	1.06035
Formic acid, LC-MS grade	Merck	VWR 84865.260
Amphetamine-d5	Mikrolab (Cerilliant)	A-013
Cocaine-d3	Mikrolab (Cerilliant)	C-014
Diazepam-d5	Mikrolab (Cerilliant)	D-910
Phenobarbital-d5	Chiron	9654.12

**Deposited data**		

Case/control ratios of preprocessed metabolomics data.	This study	Data S2
Analysis code for running models etc.	Zenodo	Zenodo: https://doi.org/10.5281/zenodo.22269885

**Software and algorithms**		

R language	R Core Team	https://www.R-project.org/(v4.4.0)
msConvert	ProteoWizard	proteowizard.sourceforge.net
XCMS	Bioconductor	https://doi.org/10.1021/acs.analchem.5c04338 v4.4.0
Glmnet	Cran	https://doi.org/10.32614/CRAN.package.glmnet v4.1-8
Caret	Cran	https://doi.org/10.32614/CRAN.package.caret v7.0-1
Ranger	Cran	https://doi.org/10.32614/CRAN.package.ranger v0.17.0
fda	Cran	https://doi.org/10.32614/CRAN.package.fda v6.2.0
metid	github.com/tidymass/metid	https://doi.org/10.1093/bioinformatics/btab583 v1.2.35
CAMERA	Bioconductor	https://doi.org/10.18129/B9.bioc.CAMERA V1.62.0

**Other**		

AcroPrep Advance filter plate	Pall Corporation	8165
Tecan Freedom EVO 200 liquid handler	Tecan Trading AG, Switzerland	N/A
Waters ACQUITY I-Class UPLC system	Waters Corporation, Milford, MA, USA	N/A
ACQUITY UPLC HSS T3 C18 column (2.1 mm × 100 mm, 1.8 μm)	Waters Corporation, Milford, MA, USA	186003539
Bruker maXis Impact QTOF	Bruker Daltonics, Bremen, Germany	N/A
Milli-Q IQ 7000	Merck	N/A

### Experimental model and study participant details

Cases included Danish Blood Donor Study participants aged 18–75 years, diagnosed with osteoporosis (ICD10-DM80 and ICD10-DM81), who had given written consent for research participation. Eligible participants had at least one sample collected within a year prior to their first diagnosis and at least 5 donations at 8 to 14 months intervals. All samples, both for cases and controls, were collected between 2010 and 2022 and had not undergone any freeze-thaw cycles. See C. Erikstrup et al. for technical details about the cohort, storage, and sample collection.^7^ See Table S2 for demographics and Table S3 for average time between sampling and diagnosis of each timepoint.

Exclusion criteria included missing data on BMI and smoking status, diagnoses (ICD and ATC codes in Table S4) of eating disorders, alcoholism, or gonad dysfunctions, and administration of osteoporosis medication during the donation period. Medications included Bisphosphonates (M05BA01, M05BA04, M05BA06, M05BA07), bisphosphonates combinations (M05BB01, M05BB03), strontiumrenalate (M05BX03), denosumab (M05BX04), raloxifene (G03XC01), teriparatide (H05AA02), and parathyroid hormone (Preotact, H05AA01). Controls were also excluded based on these criteria. This resulted in an initial cohort of 70 cases, 56 of which continued donation post-diagnosis, and 54 were diagnosed without a pathologic fracture (DM81).

Due to resource constraints, 39 longitudinal and 60 single time point cases were selected and paired with matched controls. Only longitudinal cases with 5 (23 participants) or 6 (16 participants) donations were used in the study to avoid breaking longitudinal sequences of participants with more than 6 years of samples, allowing for later validation studies. Single time point cases were selected from an initial cohort of 205 cases that met the same inclusion criteria as the longitudinal data, except they only required at least one donation back in time, still within a year of diagnosis. The 60 single time points cases were fixed to the same sex ratio as the initial longitudinal cohort (58/70 female) but were otherwise randomly selected. Controls were chosen based on the absence of osteoporosis diagnosis within one year after the latest donation and matched based on birth year, region of inclusion, sex, and donation date ( ±60 days) to ensure similar storage times. The study design comprised 540 plasma samples from 198 participants in total, of which two samples were lost during extraction (final total: 538). Matched pairs were analyzed within the same analytical batches.

#### Influence of age, sex, and ethnicity

Cases and controls were matched on age and sex, and no model included these variables as predictors, so the reported discriminative performance cannot be attributed to age/sex differences. Participants were born between 1945 and 1995 (Table S1, binned to preserve k-anonymity). The exact age at donation is not reported to avoid identifying individual participants at the extremes of the distribution. Because the cohort is predominantly female (166 of 198 participants), we were not powered to test whether the identified metabolite trajectories differ between women and men. Ethnicity was not recorded in the Danish Blood Donor Study and could therefore not be reported; the donor population is expected to be predominantly Danish/Northern European.

#### Ethics

DBDS was approved by the Scientific Ethical Committee of Central Denmark (M-20090237) and the Zealand Region (SJ-740) and by the Danish Data Protection Agency (P-2019–99). The DBDS was conducted in accordance with the ethical principles outlined in the Declaration of Helsinki.

### Method details

#### Chemicals

Acetonitrile (LC-MS grade), methanol (LC-MS grade), formic acid (FA, LC-MS grade), amphetamine-*d*_*5*_, cocaine-*d*_*3*_, diazepam-*d*_*5*_, and phenobarbital-*d*_*5*_ were acquired from Merck (Darmstadt, Germany). MilliQ water was prepared using a Milli-Q IQ 7000.

#### Plasma sample extraction

Plasma sample extraction was performed in 2 mL 96 well plates using a Tecan FREEDOM EVO 200 (Tecan Trading AG, Switzerland). In brief, 200 μL plasma were added 100 μL 0.1M HCl followed by 134 μL of an internal standard mix containing amphetamine-*d*_*5*_ (60 ng/mL), cocaine-*d*_*3*_ (60 ng/mL), diazepam-*d*_*5*_ (60 ng/mL), and phenobarbital-*d*_*5*_ (1.2 μg/mL) in methanol. The mixture was shaken at 1650 rpm for 30 s following each addition. Acetonitrile was added three times at a volume of 67 μL and shaken 30 s in between. Finally, 200 μL acetonitrile was added, and the mixture was left to stand for 15 min. The sample was centrifuged at 5000xg for 5 min and 500 μL was transferred to an AcroPrep Advance 96 Well 1 mL 30K Omega filter plate (Pall Corporation, NY, USA). The filter plate was centrifuged at 2000xg for 5 min and the filtrate was transferred to a 1 mL glass insert in a 96 well plate that was evaporated under a stream of nitrogen. The pellet was redissolved in 100 μL MilliQ water containing 5% acetonitrile and 0.1% FA.

#### LC-HR-MS analysis

The samples were subject to untargeted metabolomics analysis using an ACQUITY I-Class UPLC system (Waters Corporation, Milford, MA, USA) coupled to a Bruker maXis Impact QTOF mass spectrometer (Bruker Daltonics, Bremen, Germany). The mass spectrometer was operated in both positive and negative electrospray ionization (ESI) mode. Chromatographic separation employed an ACQUITY UPLC HSS T3 C18 column (2.1 mm × 100 mm, 1.8 μm, Waters) with gradient elution. Mobile phase A comprised MilliQ water with 0.1% FA, while mobile phase B consisted of acetonitrile with 0.1% FA. The column temperature was maintained at 50°C, with a flow rate of 0.4 mL/min. The gradient elution commenced at 0% B (0-2min), followed by linear increases to 40% B (2-6min), 60% B (6-6.5min), 88% B (6.5–11min), 100% B (11-11.5min), held at 100% B (11.5–17min), linear decrease to 0% B (17-18min), and finally, equilibration at 0% B (18-21min). The injection volume was 5 μL in both positive and negative ESI. The temperature in the autosampler was kept at 6°C.

MS scans were conducted in full scan mode at a sampling rate of 4Hz across a mass range of 50–1000 m/z. The nebulizing gas pressure was maintained at 1.2 bar, with a capillary voltage of 4.0 kV in positive ESI and 2.5 kV in negative ESI. The drying gas flow rate was 8.0L/min at a temperature of 220°C. MS/MS scans using data-dependent acquisition (auto-MS/MS) followed similar parameters, with collision energies set at 10, 20 or 30 eV and a sampling rate of 10Hz. Internal calibration was conducted at the conclusion of each run using sodium formate in both ESI modes.

The samples were extracted and analyzed in seven batches, with all time-series samples from the same participant analyzed within the same batch. Matched cases and controls were additionally analyzed within the same batch. A control sample containing 37 standard compounds was analyzed at the beginning and end of each batch for quality assessment of instrument performance. The system was equilibrated with four injections of a separate blood sample not part of the study (QC-sample). The QC sample was additionally injected for every 10^th^ sample during each batch to ensure instrument stability during the run. Samples within a batch were analyzed in randomized order. Eight samples within a batch were analyzed twice as short technical replicates. Additionally, four samples from a previous batch were analyzed within each new batch as batch replicates.

A sample containing 20 different pooled samples from the study were used for MS/MS fragmentation analysis for annotation/identification of metabolites.

### Quantification and statistical analysis

#### Pre-processing of LC-MS data

The mass spectrometry data from UPLC-HR-QTOF were converted to *mzML* file format using msConvert from ProteoWizard (http://proteowizard. sourceforge.net). XCMS was used to create the feature table from the semiquantitative untargeted LC-MS data.^33^ Here, Centwave (ppm = 12, snthresh = 6, peakwidth = 4–25) was used for peak identification, peak density (min fraction = 0.3) was used for peak grouping and CAMERA was used for isotope annotation. Imputation was performed with the XCMS fillChromPeaks method that integrates the baseline signal/noise in samples where no peak was detected. In rare cases with no baseline signal, zero was used for imputation. Metid (part of tidymass R package^34^) was used to automatically annotate the MS/MS scans, at level 2 identification according to the Metabolomics standard initiative.^35^ The following databases were used: Massbank, Mona, NIST, msdatabase (Snyder lab), HMDB, orbitrap, and the Fiehn Hilic database. Automatic matches were manually evaluated and compared against an in-house database for level 1 identification when available. The *total_score* threshold was set to 0.5, which was a weighted scoring of precursor m/z match(0.5) and the ms2 dotproduct(0.5) for public libraries and for the inhouse matching weights of 0.25, 0.25, and 0.5 were used for precursor m/z match, retention time match, and ms2 dotproduct. All annotated compounds discussed in the study have been manually assessed by matches in ms2 fragments to libraries (Figure S11).

#### Statistical preprocessing

The data were log-transformed, and features with more than 10% missing values were removed. Next, features were processed with robust row normalization, by dividing rows (samples) by the row sums of a subset of robust features. The robust features had median intensities between the 20 and 80 quantiles of all feature medians and min-max intensity differences between the 20 and 80 quantiles of all feature min-max differences. This ensured that highly fluctuating features did not contribute to normalizing the matrix effects (i.e., systematical differences across all intensities). The features were then normalized using a setup inspired from Ritchie et al.^36^ that used stepwise regressions to subtract systematical noise signal from the data. In our normalization all corrections were done simultaneously using the following model:

$$Int_{conf}=Region+Batch+InjOrder+Storage+batch\cdot InjOrder+batch\cdot Storage$$

$$Intensity_{corrected}=Intensity-Int_{conf}$$

Where Int_conf_ is the intensity explained by confounders, InjOrder is the injection order during an LC-MS batch, and Intensity_corrected_ is the corrected intensity after regressing out the confounding variables. In this two-step normalization, the row-normalization normalizes potential sample-related technical noise, and the regression-based normalization normalizes systematical confounders.

After normalization, features were removed based on technical replicate correlations (remove if correlation coefficient <0.8) and quality control relative standard deviation (RSD) (remove if > 0.1). Features that correlate strongly across technical replicates are unlikely to reflect random noise, signal in blinds (water extracted as samples), or carry-over between samples (Figure S12). High QC RSD indicates batch effects and combining both filters removes features most susceptible to technical variance (Figure S13). Blinds were run in the experiment, and the statistical analyses were tested with and without use of blind filters (blind mean <1% sample mean and blind SD < 1% sample SD) without any effect on the results (see Methods S1 for additional details). For positive ESI, 8851 features were removed, and for negative ESI, 3047 features were removed, resulting in 3221 and 1429 features. The datasets were analyzed individually, as we expected collinearity would increase substantially upon concatenation and reduce interpretation of feature importance.

#### Representing longitudinal data

The slopes of the longitudinal data were calculated for all selected features in each participant where technical replicates were included. Hence, each slope was based on feature intensities at five to six time points for each longitudinal participant. Functional data analysis (FDA) was implemented with the fda R package^37^ using 4 basis B-spline functions and extracting the first principal component for each feature - these components were then used for lasso modeling.

#### Modeling

Models were trained in three settings; (1) normalized feature intensities from the latest donation before diagnosis (or no diagnosis), (2) feature slopes calculated from the longitudinal data, and (3) feature-wise PCA scores from FDA.

In each setting, a linear lasso logistic regression (glmnet R package) and a random forest model (caret and ranger R package) were hyperparameter tuned and evaluated.^38^^,^^39^^,^^40^ The glmnet models were tuned using the default settings (glmnet.cv) and the ranger models were tuned using a train length of 5 in caret. The Random Forest parameters provided by caret were: *mtry* of [2, 10, 53, 276, 1428] for negative ESI and [2, 12, 80, 508, 3220] for positive ESI, *min.node.size* of [1], and *splitrules* of [*gini*, *extratrees*]. See Data S3 for hyperparameter tuning results and specific lambda values for the glmnet models. Leave-one-pair-out-cross-validation ensured matched pairs stayed together during evaluation.

#### Model parameters

For all models, custom cross-validation was used to assign matched pairs to the same test-folds. For the cross-sectional models, the optimized *lambdas* were 0.050 in the negative ESI model, and 0.077 in the positive ESI model. For Random Forest the best hyperparameter set for the negative ESI model was *mtry* of 276 using *gini* as splitrule and for the positive ESI model the best *mtry* was 3220 using *gini* as splitrule.

For the slope lasso model, the optimal lambda values were 0.030 for the negative ESI model and 0.018 for the positive ESI slope model. For FDA the lambdas were 0.015 and 0.16 for negative and positive ESI. For slopes, the random forest hyperparameter tuning yielded an optimal *mtry* of 1428 and *splitrule* of *extratrees* for the negative ESI model and *mtry* of 508 and splitrule of *gini* for the positive ESI model. For FDA, the Random Forest hyperparameter tuning yielded the same results.

#### Technical validation and ablation tests

To perform an evaluation of technical replicability, the slope models were also evaluated using leave-one-batch-out cross-validation. Here, one batch was held out for evaluation during training on the other batches. Technical replicates spanning across batches were not included in training to avoid any data leakage during training.

The best performing model (lasso on slopes from negative ESI data) was further tested in two different settings. The sample size was gradually decreased by one matched longitudinal pair at a time (5–6 case samples and 5–6 control samples) until the total longitudinal sample count matched that of the cross-sectional model (198 samples) in order to assess model performance under an equivalent total data budget. At each iteration the models were fitted and evaluated using the same methods as the approach used on the full dataset. For the other test, leave-one-matched-pair out models (*n* = 39) were evaluated on hold-out pairs with gradually diminishing longitudinal sequences. In each iteration the sample closest (across the full dataset) to diagnosis was removed including its matched pair and the new test data was evaluated (leave-one-matched pair out but with one sample less to calculate slopes). Samples were removed until the first matched pair reached a longitudinal basis of less than two samples per participant, as this impeded slope calculation. This approach illustrates a deployment scenario where the real-world test data does not follow the same structure as the training data.

#### Model evaluation

ROCs were created to assess the performances for the latest sample model, the slope model, and the FDA model. In-depth analysis of certainties at different decision thresholds was limited by power (sample size) as single samples would contribute to large discrete jumps in performance.

AUCs were calculated based on the ROCs of each model and confidence intervals were estimated by bootstrapping of the model predictions (1000 resampled runs). The standard deviation of the bootstrapping result was used to determine the AUC standard error which was used to calculate the CI (AUC ±1.96∗SE). Lastly, the batch validation and temporal robustness models were evaluated with *p*-values calculated from the binomial distribution, using the number of correct class predictions (test statistic) as successes vs. the observed frequency as null hypothesis (automated by caret).^38^

#### Feature selection

The feature importance was extracted from the best model from the lasso logistic regression slope models of positive and negative ESI datasets (Data S1). The importance was represented as the median coefficient from each cross-validation training fold to ensure robustness and stability. The median coefficient was used to support that noisy predictors would get an importance of 0.

## Supplemental information

Document S1. Figures S1–S13 and Tables S1–S4

Data S1. Feature importance in negative and positive ESI data

Data S2. Anonymized case-control ratios of feature table

Data S3. Hyperparameter tuning results of glmnet models

Data S4. PCA data for Figure 1

Data S5. ROC data for Figure 2

Data S6. Boxplot data for Figure 2
