# Supplementary material for: Longitudinal pre-diagnostic blood samples predict osteoporosis risk
Source: iScience. 2026 Sep 22;29(11):117737. doi: 10.1016/j.isci.2026.117737 (PMC13659679; doi:10.1016/j.isci.2026.117737)
Supplement: Supplementary file 1 [file mmc1.pdf]

iScience, Volume *29*

Supplemental information

Longitudinal pre-diagnostic blood samples predict osteoporosis risk

Johan K. Lassen, Kirstine L. Nielsen, Lotte Hindhede, Susan Mikkelsen, Bertram Kjerulff, Sisse Rye Ostrowski, Erik Sørensen, Christina Mikkelsen, Ole Birger Vesterager Pedersen, Mie Topholm Bruun, Bitten Aagaard, Mogens Johannsen, Christian Erikstrup, and Palle Villesen

Supplementary information

Table of content

Methods

Methods S1: Quality assurance of features (feature filtering)

Figures

Figure S1: PCA of technical variance and known confounding effects.
Figure S2: PCA of normalized data.
Figure S3: PCA of osteoporosis signal.
Figure S4: Donor variance in PCA.
Figure S5: Random Forest performance in osteoporosis prediction.
Figure S6: Ablation tests of the slope lasso model in negative ESI data
Figure S7: Hold-one-batch-out cross validation demonstrates stable methodology.
Figure S8: Longitudinal slopes of Hippurate in pos and neg ESI data.
Figure S9: Hippurate abundance versus age, colored by group.
Figure S10: ROC curves based on logistic regressions of the top five predictors (pos and neg).
Figure S11: Annotation data
Figure S12: Replicates of the same sample plotted against each other per feature.
Figure S13: Distribution of QC RSD vs. technical replicate correlation

Tables

Table S1: Additional performance metrics of lasso models.
Table S2: Cohort statistics.
Table S3: Mean time from sample to diagnosis in longitudinal participants
Table S4: Exclusion criteria for donors, cases, and controls.

Methods S1 | Quality assurance of features (feature filtering)

We used a combination of correlation between technical replicates and QC RSD to filter noisy features. Figure S1 and S2 help describe this approach.

Features that correlate strongly in within-batch technical replicates are unlikely to reflect random noise, signal in blinds (water extracted as samples), or carry-over between samples. This unsupervised approach selects features with biological variation, since features with no biological variation will not correlate between replicates (random technical variation dominates instead) and are therefore uninformative for any type of differential analysis. The one exception is when batch effect itself drives the correlation, which can be addressed by applying the QC RSD (relative standard deviation) filter, excluding features where the QC RSD between batches varies by more than 10% (Fig. S2). QC RSD alone is not as effective at removing carry-over or signal that is also present in blinds, which is why it is combined with the replicate-correlation filter.

As orthogonal validation of this filtering approach, we additionally tested a blind-based filter, removing features with less than 1% standard deviation and 1% mean abundance in analytical blinds relative to samples. This additionally removed 1376 of 3221 features in positive ESI and 638 of 1429 features in negative ESI. Re-analyzing the data with this additional filter did not degrade model performance (AUC increased marginally to 0.80 in negative ESI, from 0.76, and to 0.68 in positive ESI, from 0.67), and the top 5 features by importance were unchanged in each polarity. We therefore conclude that residual blind-derived signal does not affect the study's conclusions and did not adopt blind filtering as a primary step.

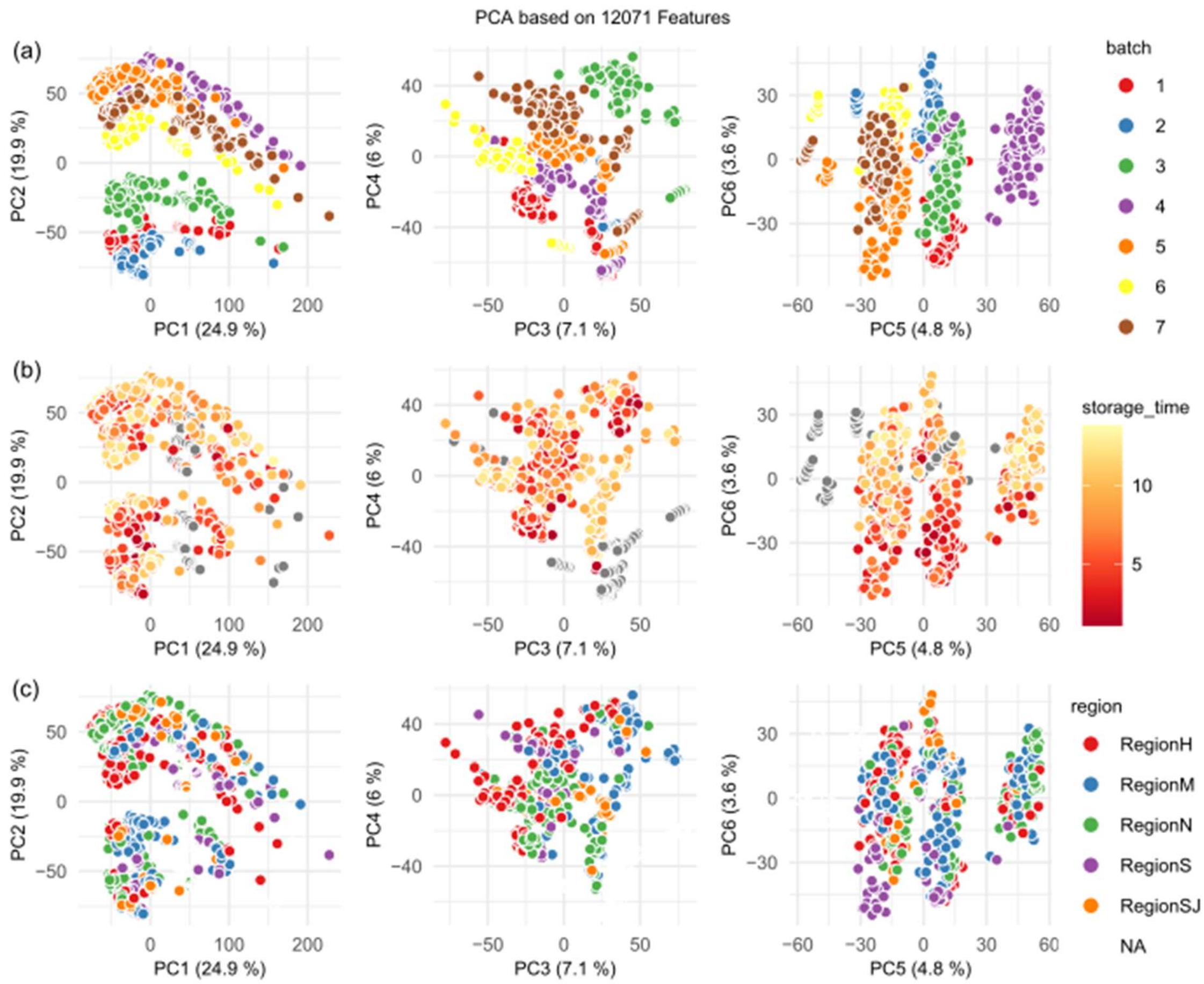

Figure S1: PCA of technical variance and known confounding effects. Positive ESI data.

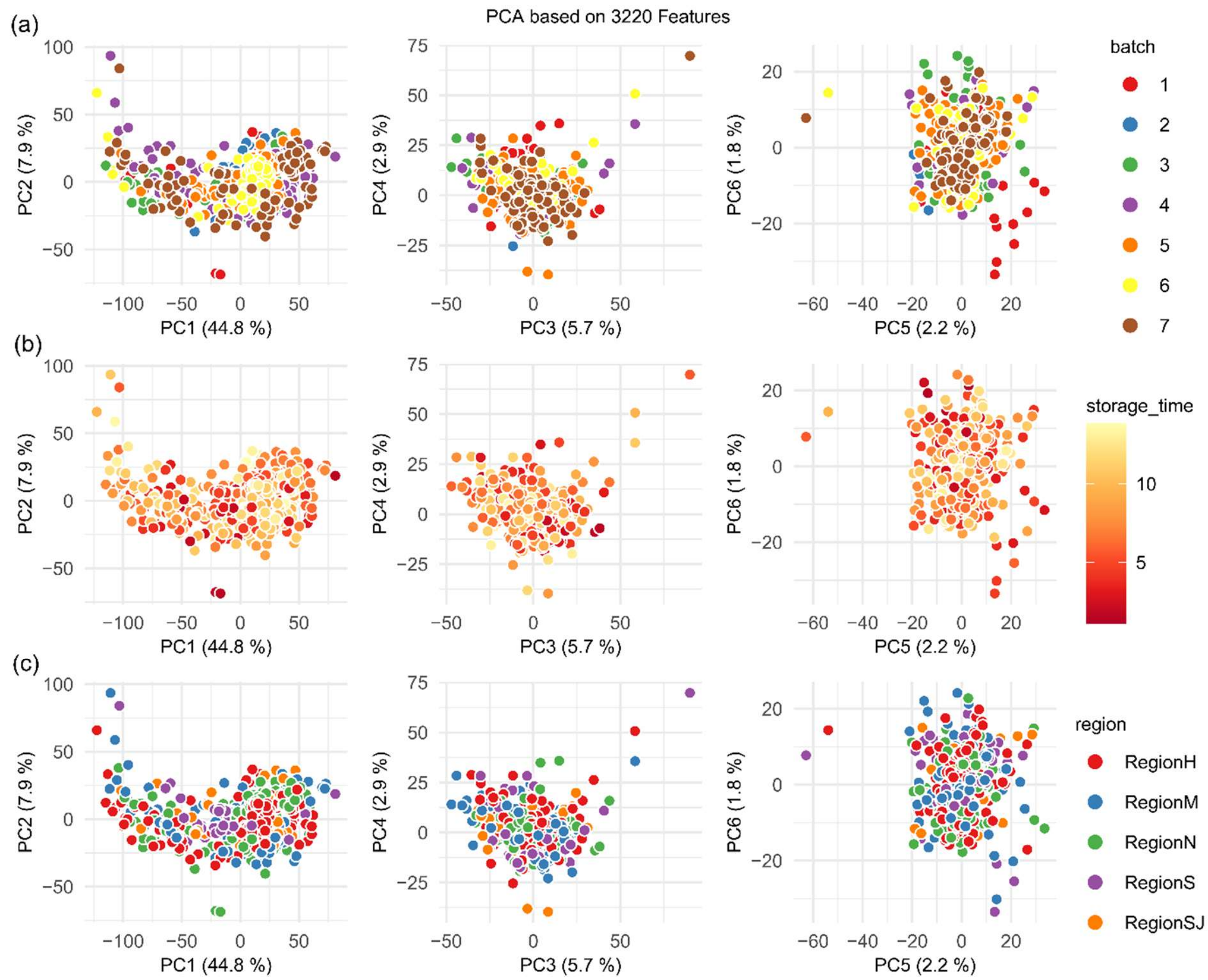

Figure S2: PCA of normalized data. Positive ESI data.

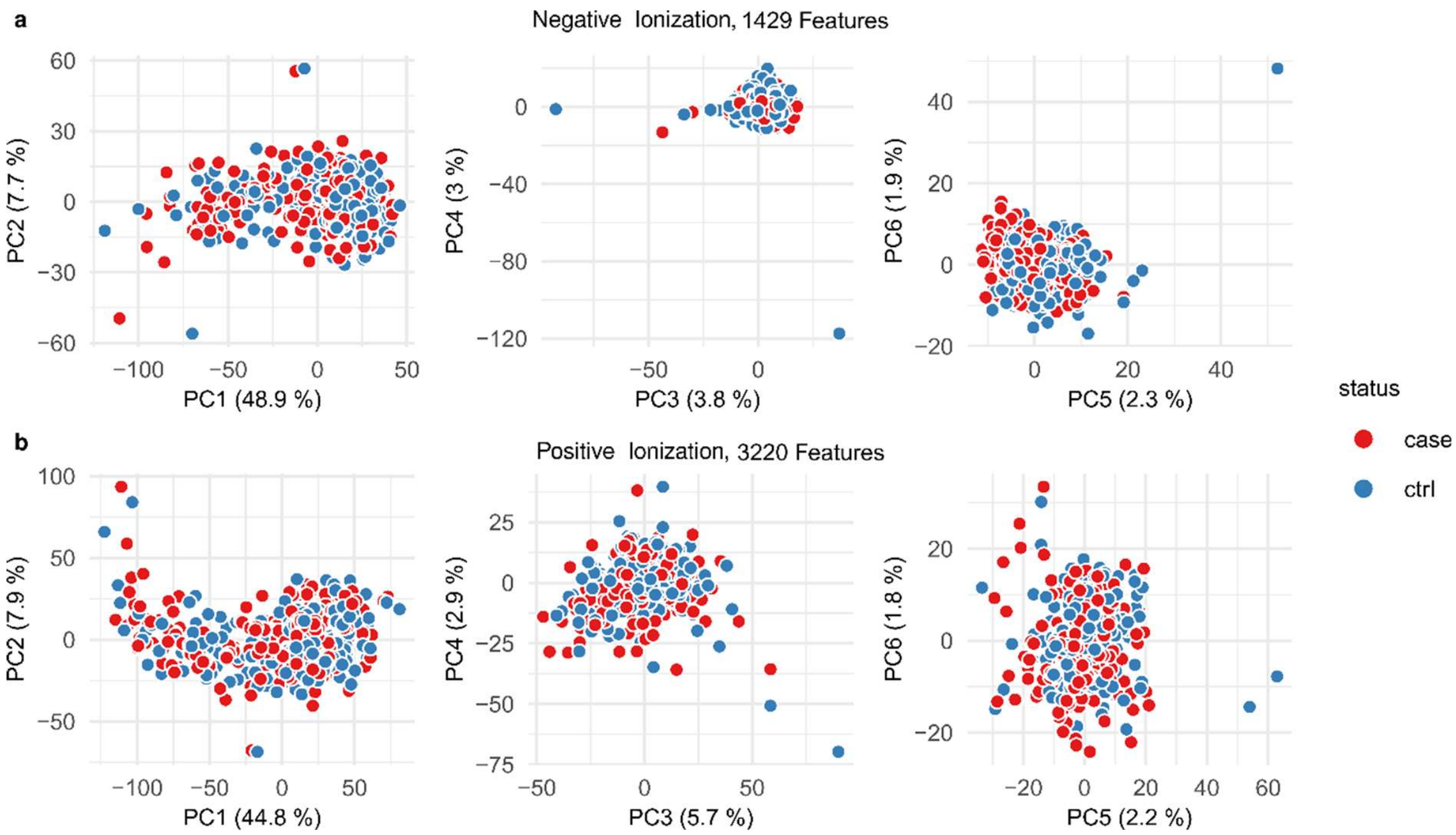

Figure S3 | PCA of osteoporosis signal. First 6 principal components of negative and positive ESI for the full dataset before calculating slopes.

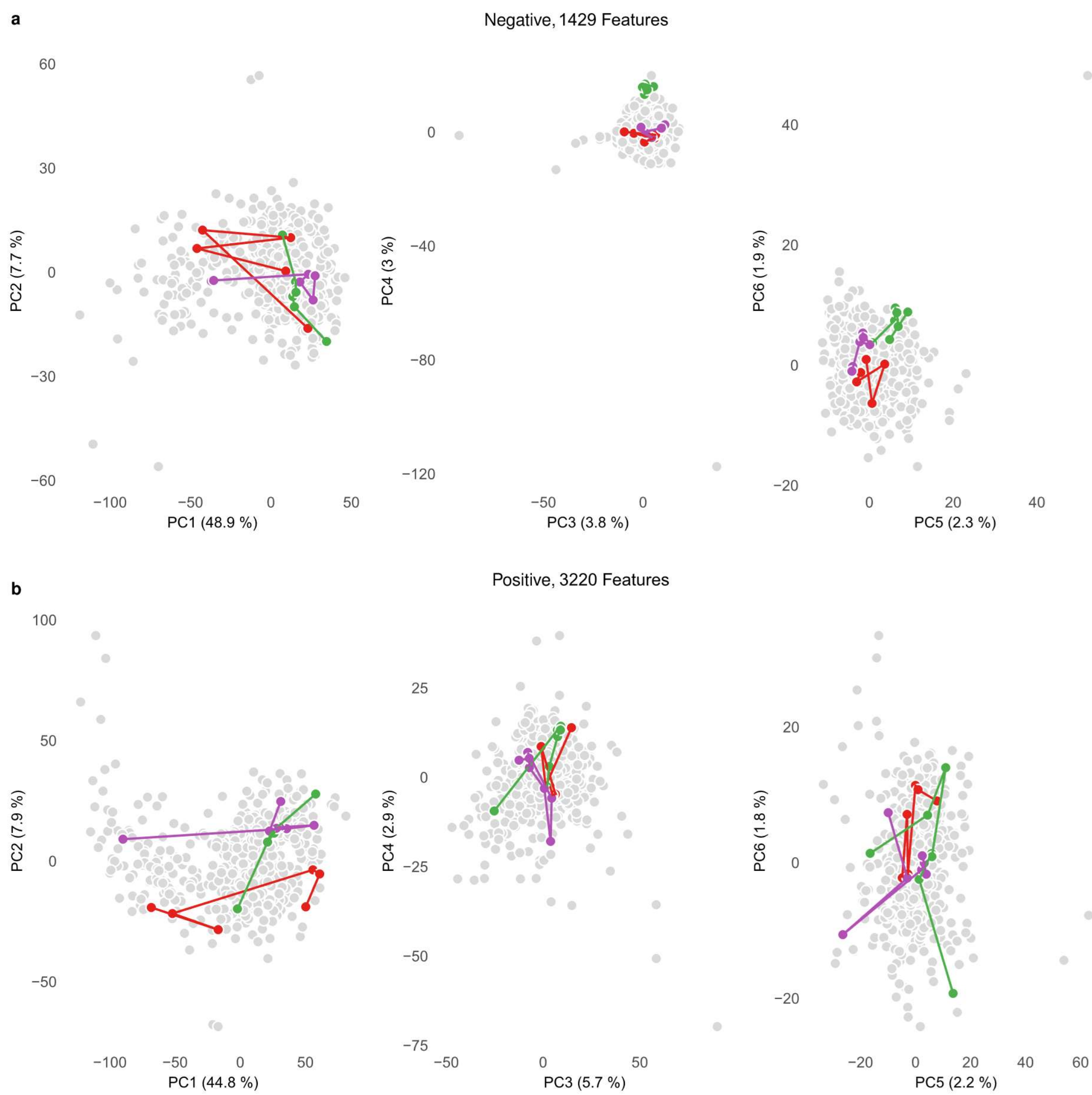

Figure S4 | Participant variance in PCA. **a.** negative ESI data colored by samples from three random participants. The first two components show that the major sources of variance are not caused by individual effects. The next four components show some clustering on individual participants. **b.** The same plot for positive ESI data. Here no participant clusters are seen likely because of more features resulting in spurious correlations and random noise.

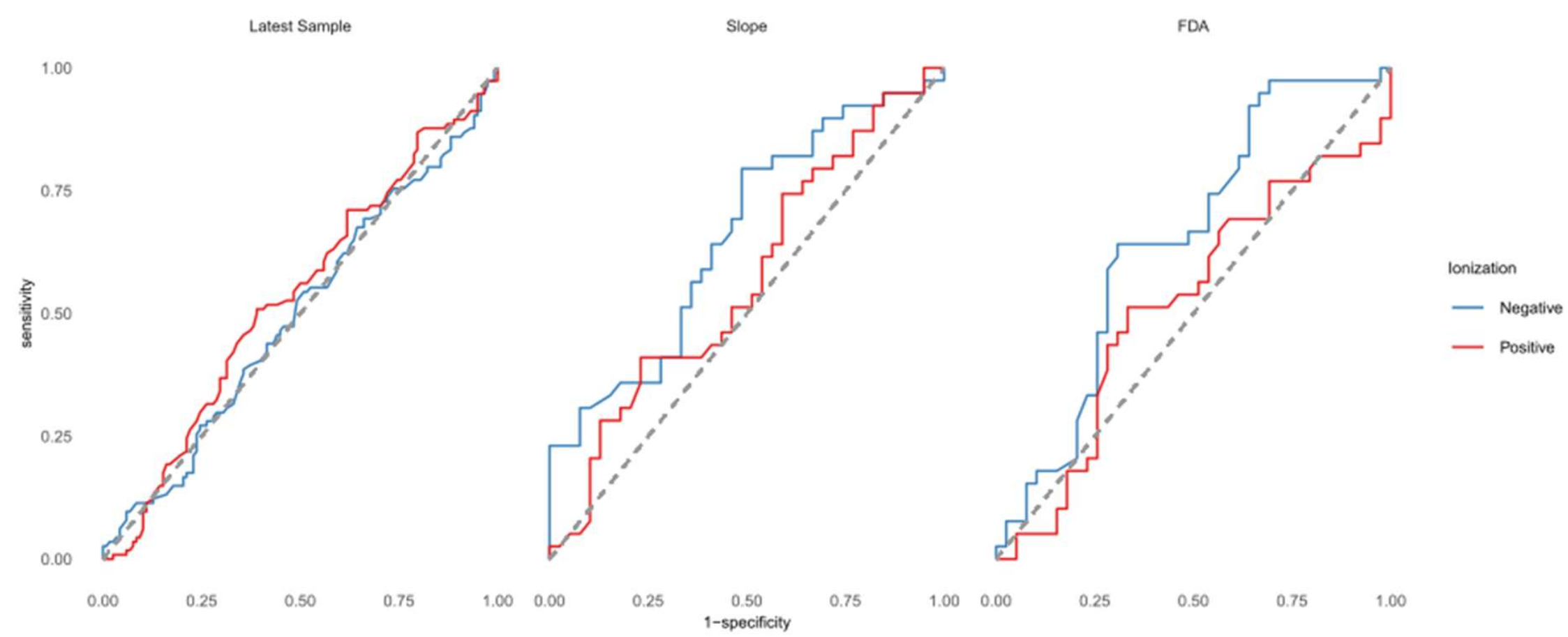

Figure S5 | Random Forest performance in osteoporosis prediction. All random forest results are non-significant.

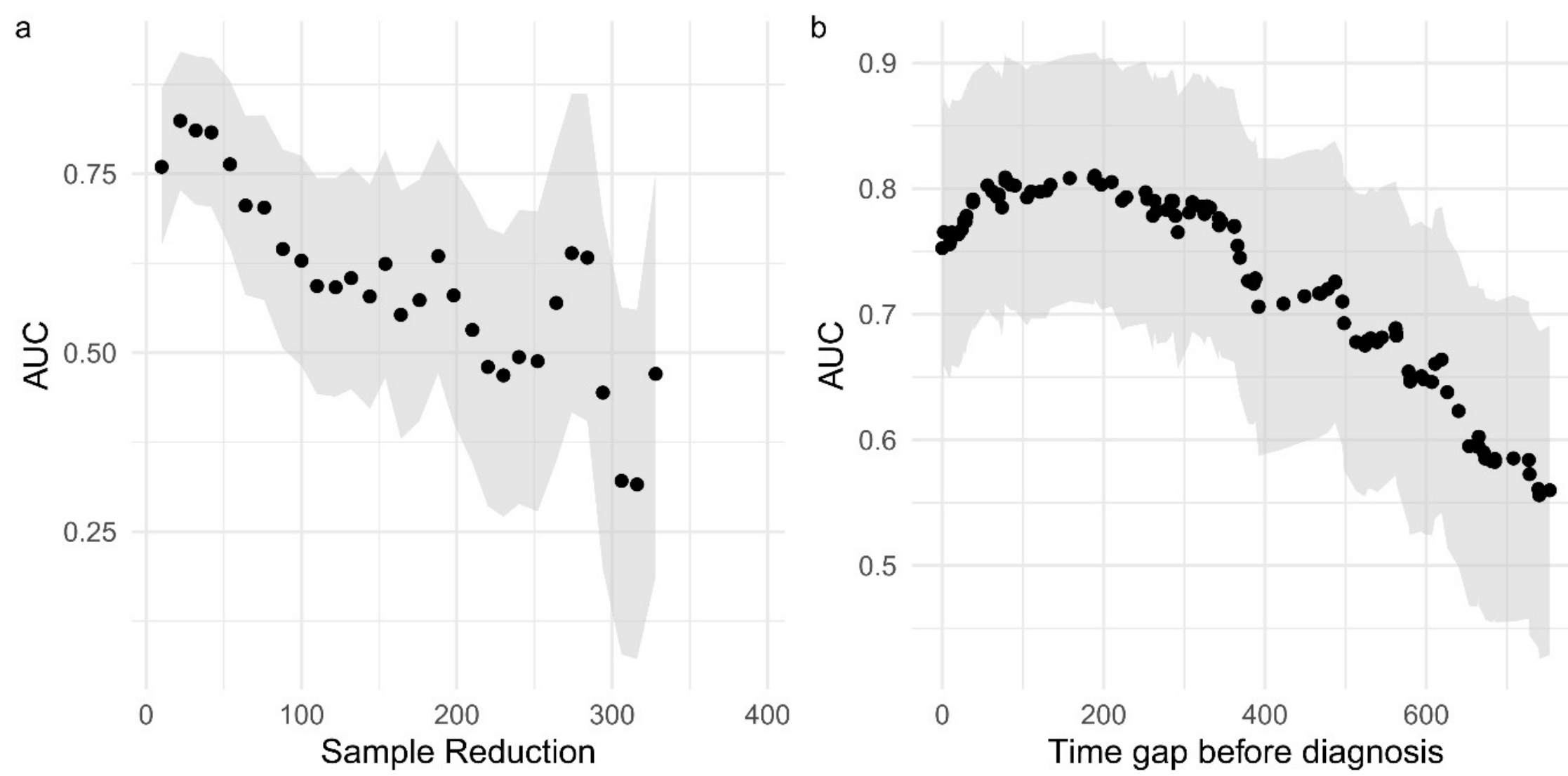

Figure S6 | Ablation tests of the slope lasso model in negative ESI data. (a) Reducing the number of samples by removing one matched longitudinal pair at a time (5-6 cases and 5-6 controls, 10-12 samples total removed per point). (b) Increasing the latest sample-diagnosis gap one sample at a time to assess robustness of the longitudinal slopes. Shaded areas represent SE calculated by bootstrapping.

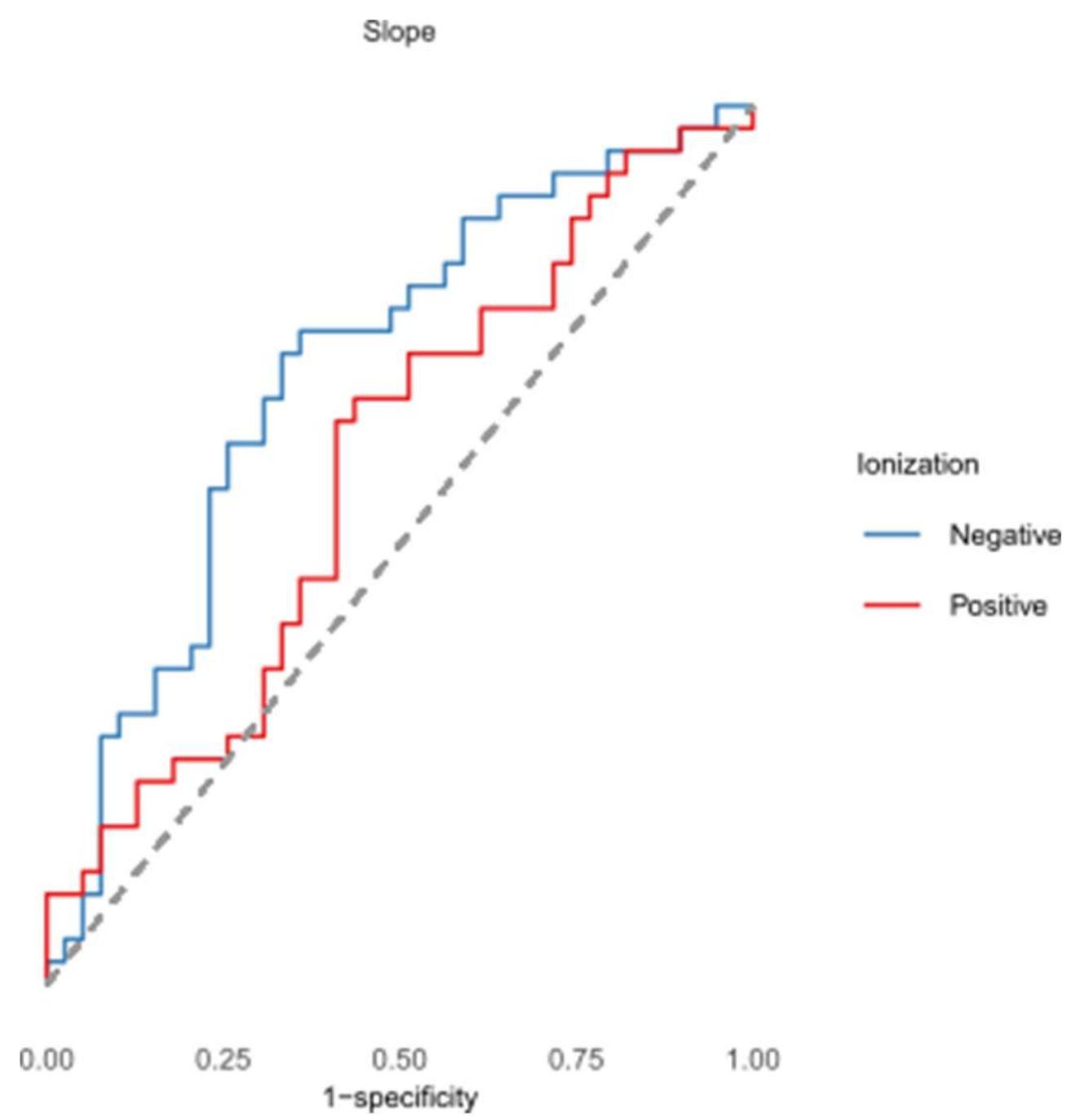

Figure S7 | Hold-one-batch-out cross validation demonstrates stable methodology. Only models of slope values (longitudinal data). The elastic net on negative ESI data validates, while the positive ESI data most likely becomes too underpowered. The AUCs are 0.59 and 0.70 for positive and negative ESI.

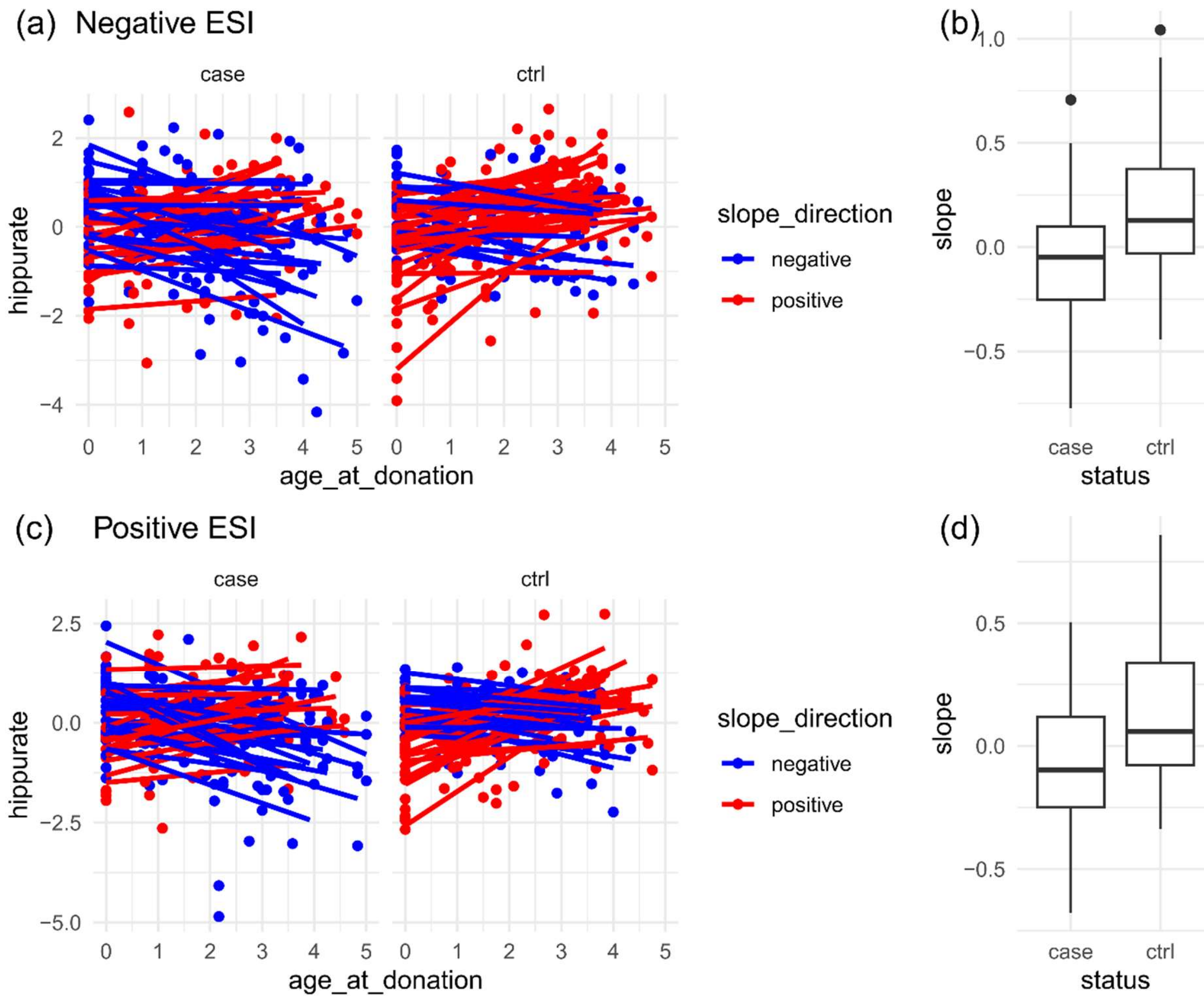

Figure S8 | Longitdudinal slopes of Hippurate in pos and neg ESI dataS. (a,c) Colored by slope (>0 or <0). (b,d) boxplots of slopes from each group.

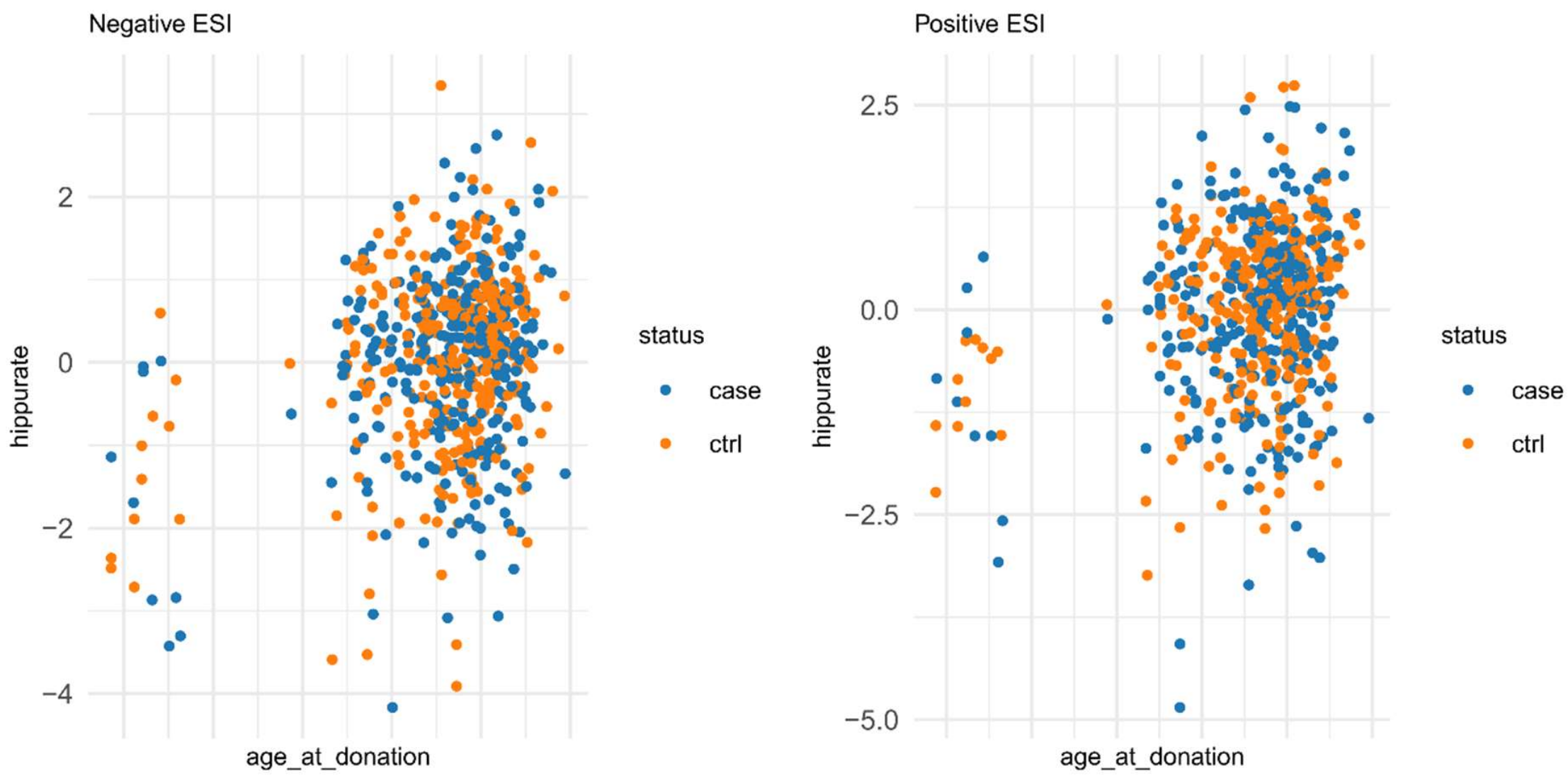

Figure S9 | Hippurate abundance versus age, colored by group. Hippurate correlation has a Pearson r of 0.25 (Neg ESI) and 0.22 (Pos ESI), corresponding to p-values of 4.2e-10 and 3.7e-8, respectively. Age does not confound Hippurate based osteoporosis predictions.

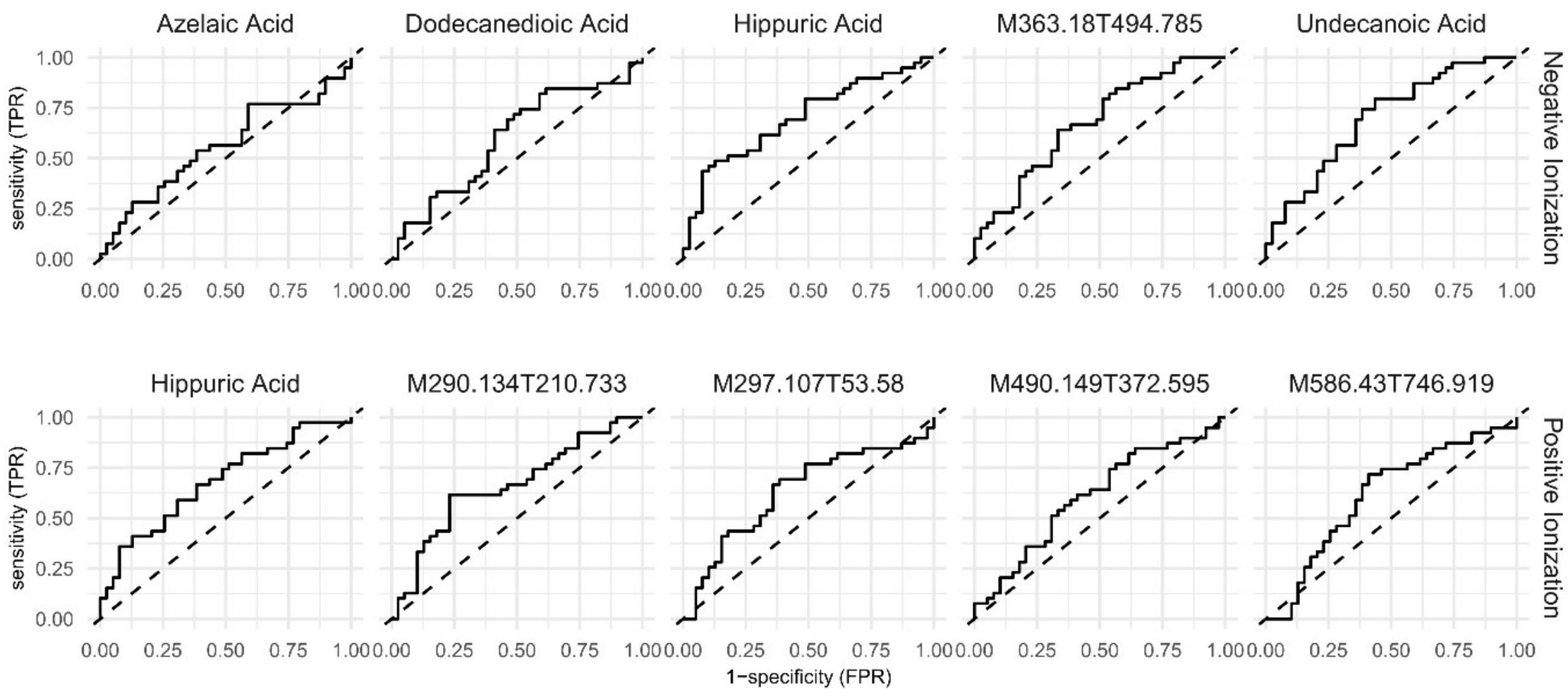

Figure S10 | ROC curves based on logistic regressions of the top five predictors (pos and neg). Hippuric acid displays a consistent predictive behavior (albeit weak).

Figure S11 | Annotation data. Chromatograms and butterfly spectra of all features presented in main text. The figure spans multiple pages until next figure title.

Chromatogram: Dodecanedioic acid (top) + adduct (bottom), ESI-

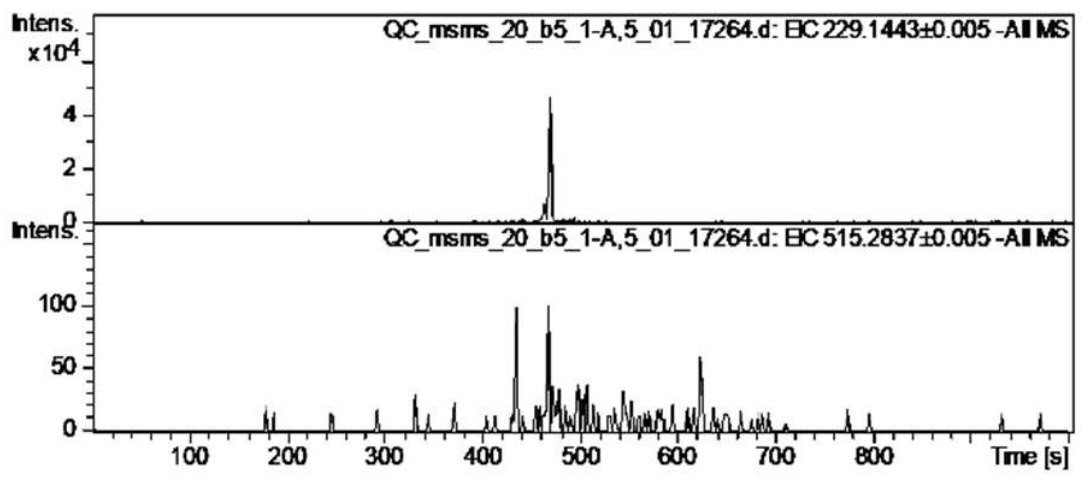

MS2 library match

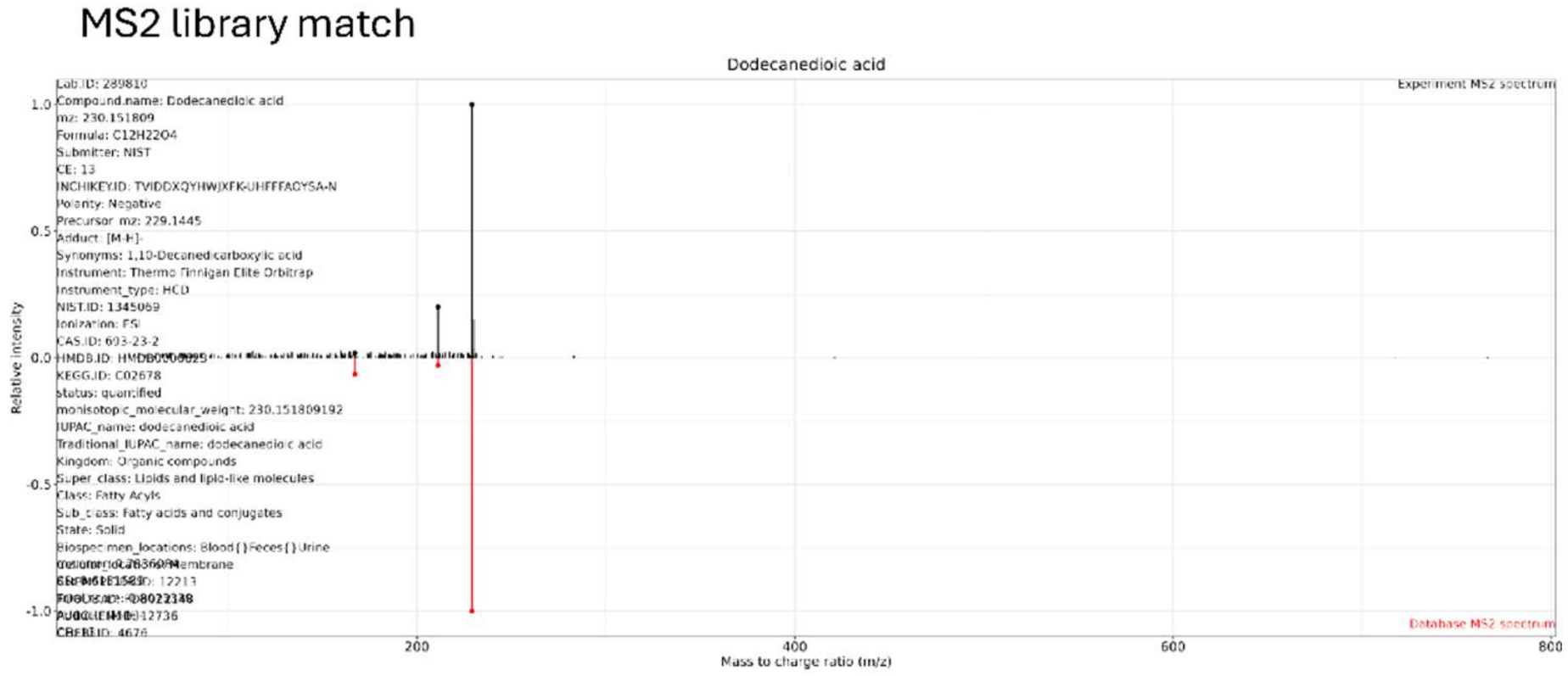

Chromatogram: Hippuric acid (top) + adduct (bottom), ESI-

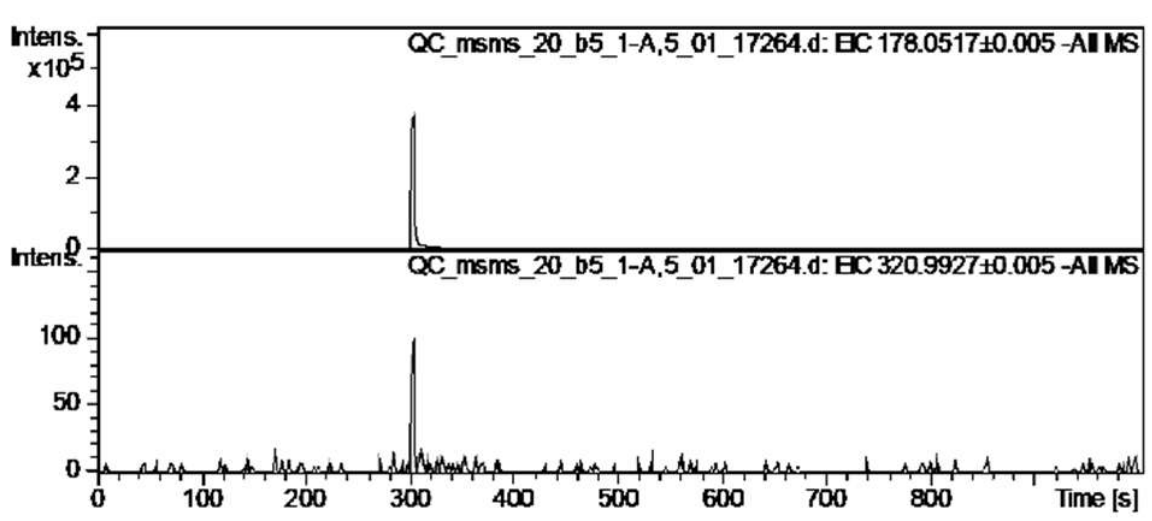

MS2 library match

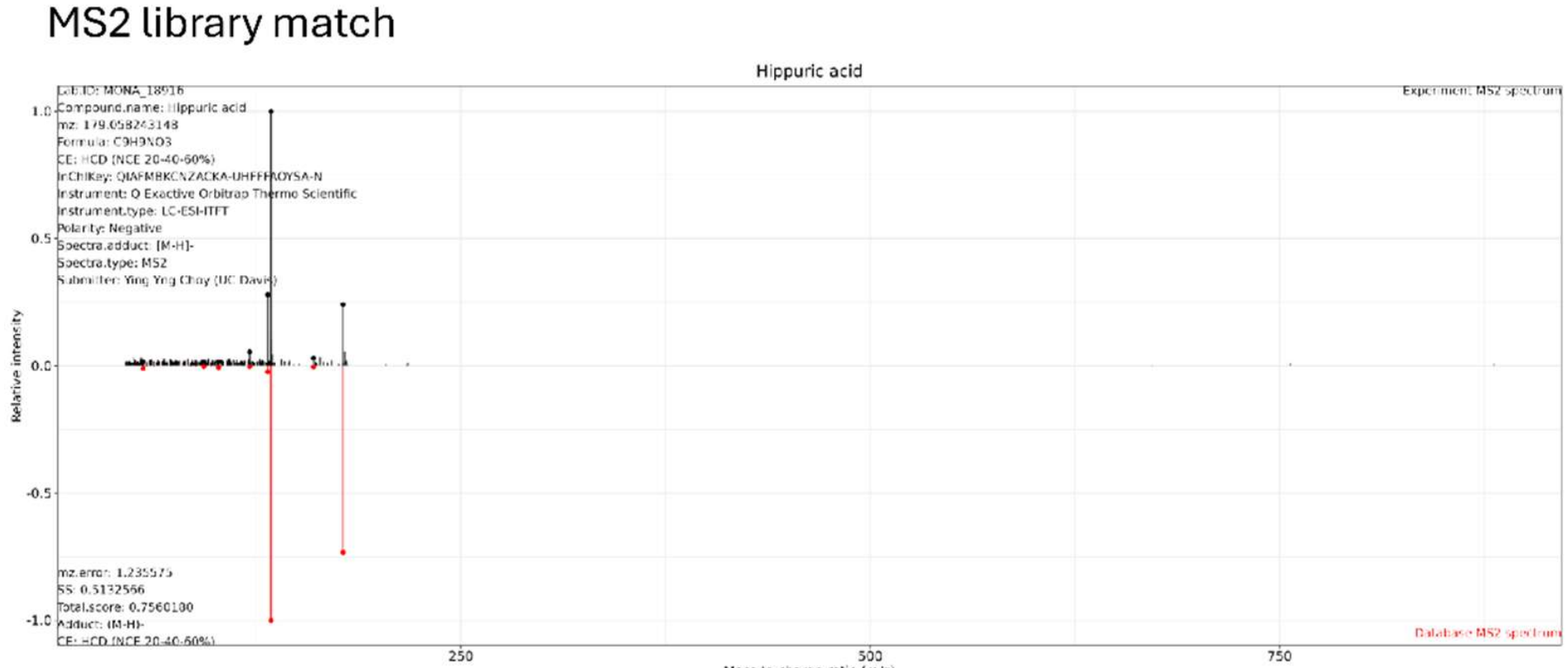

Chromatogram: Undecanoic acid (top) + adduct (bottom), ESI-

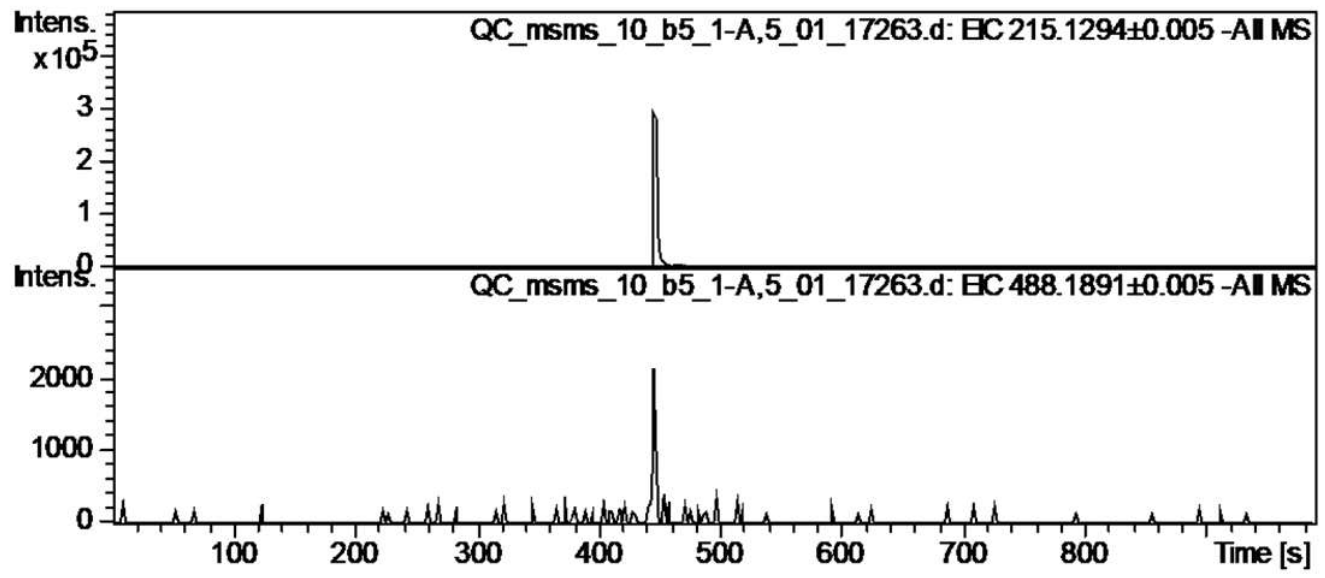

MS2 library match

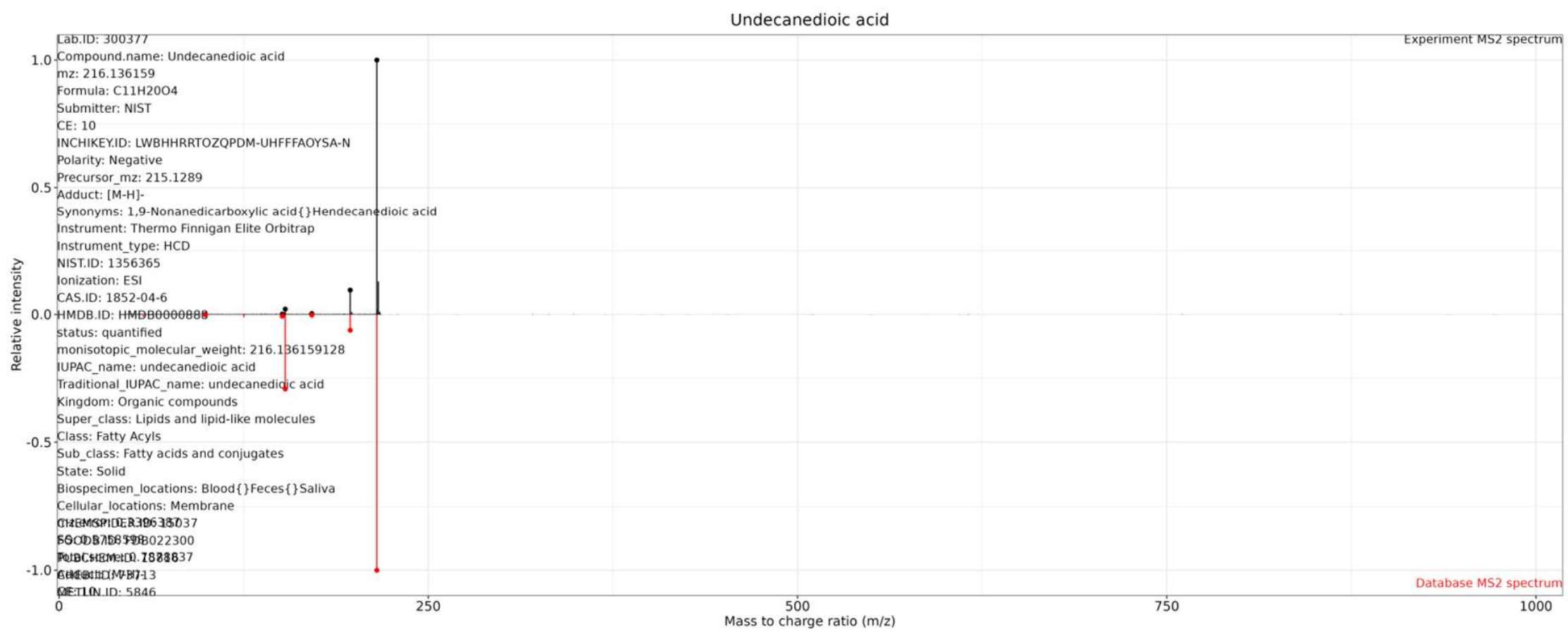

Chromatogram: Azelaic acid (M-H2O-H), ESI-

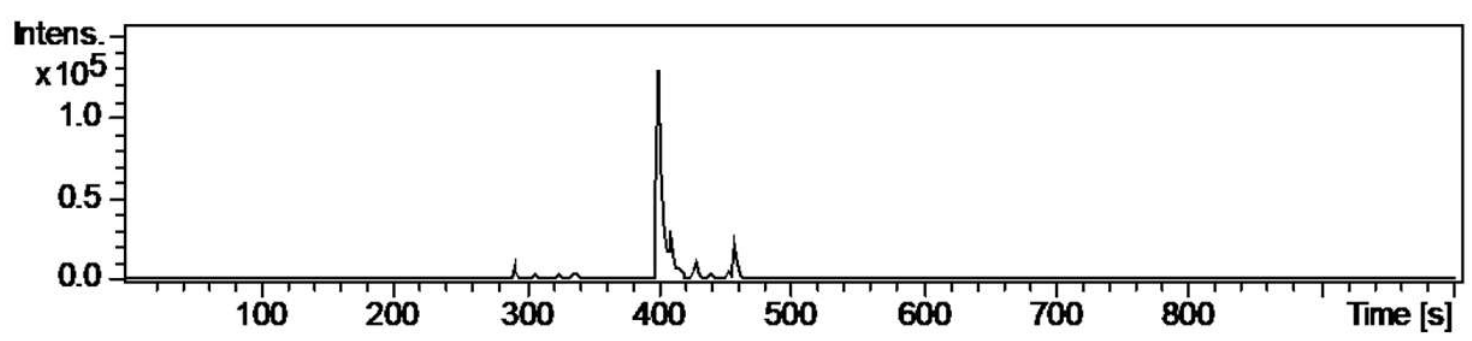

MS2 library match

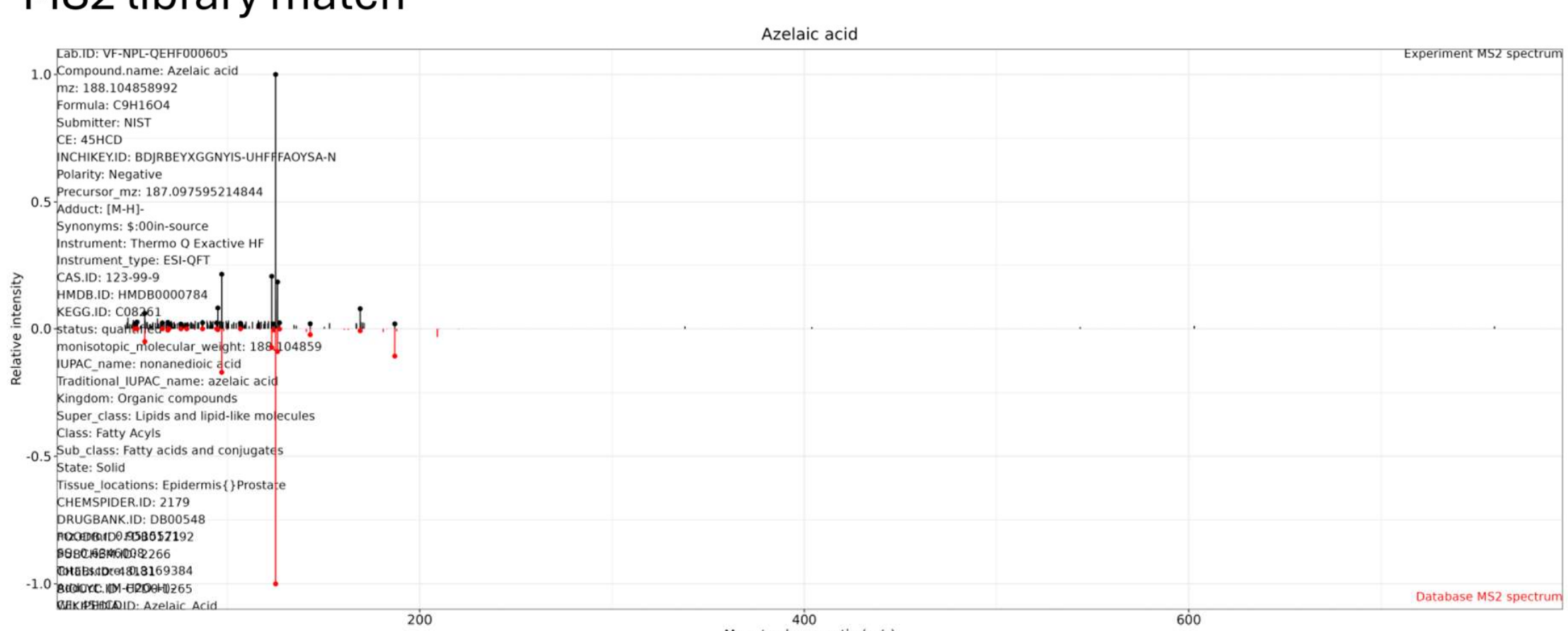

Chromatrograms: Hippuric acid (top) + adduct (bottom) [M+3]+, ESI+

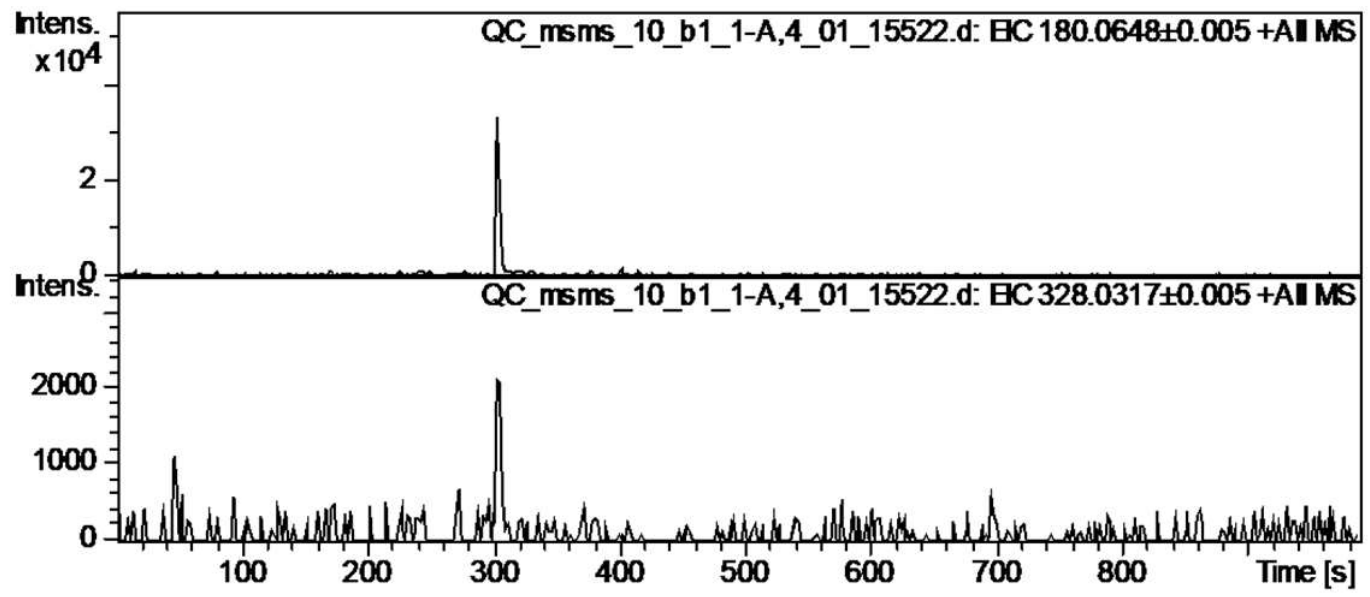

MS2 library match

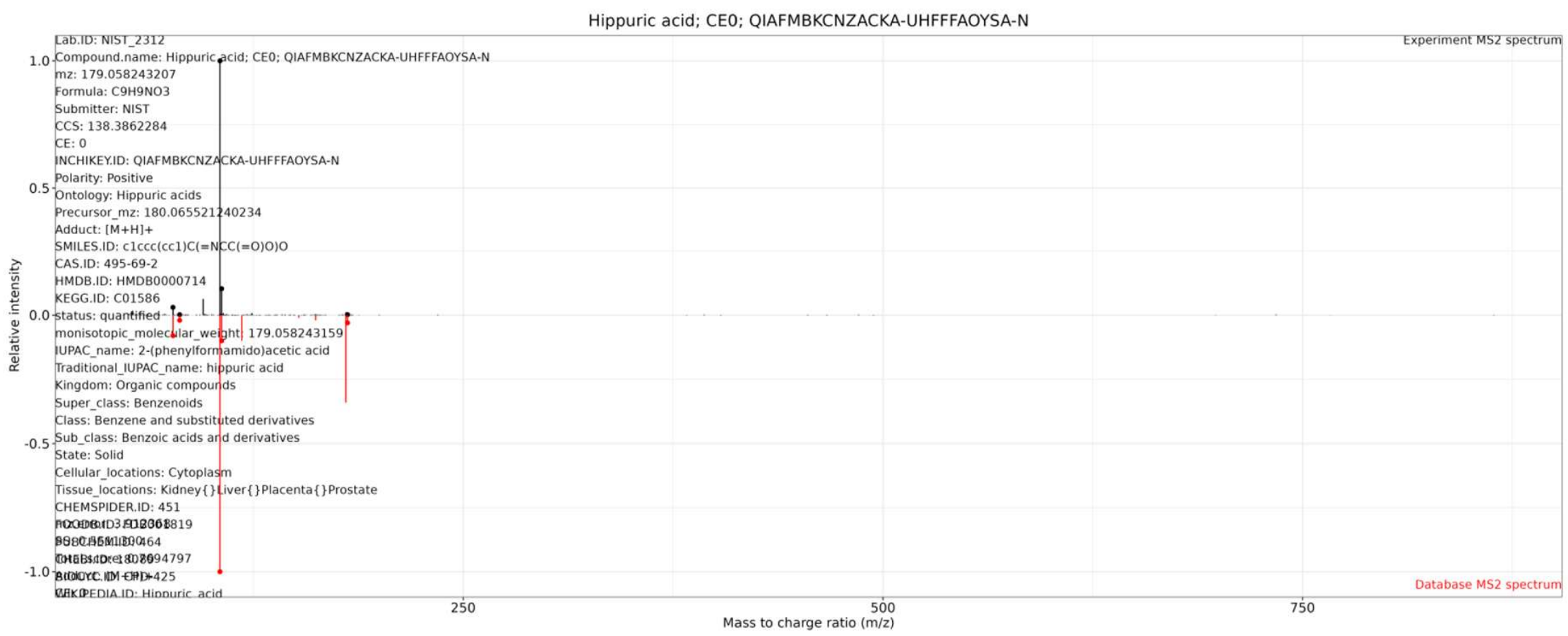

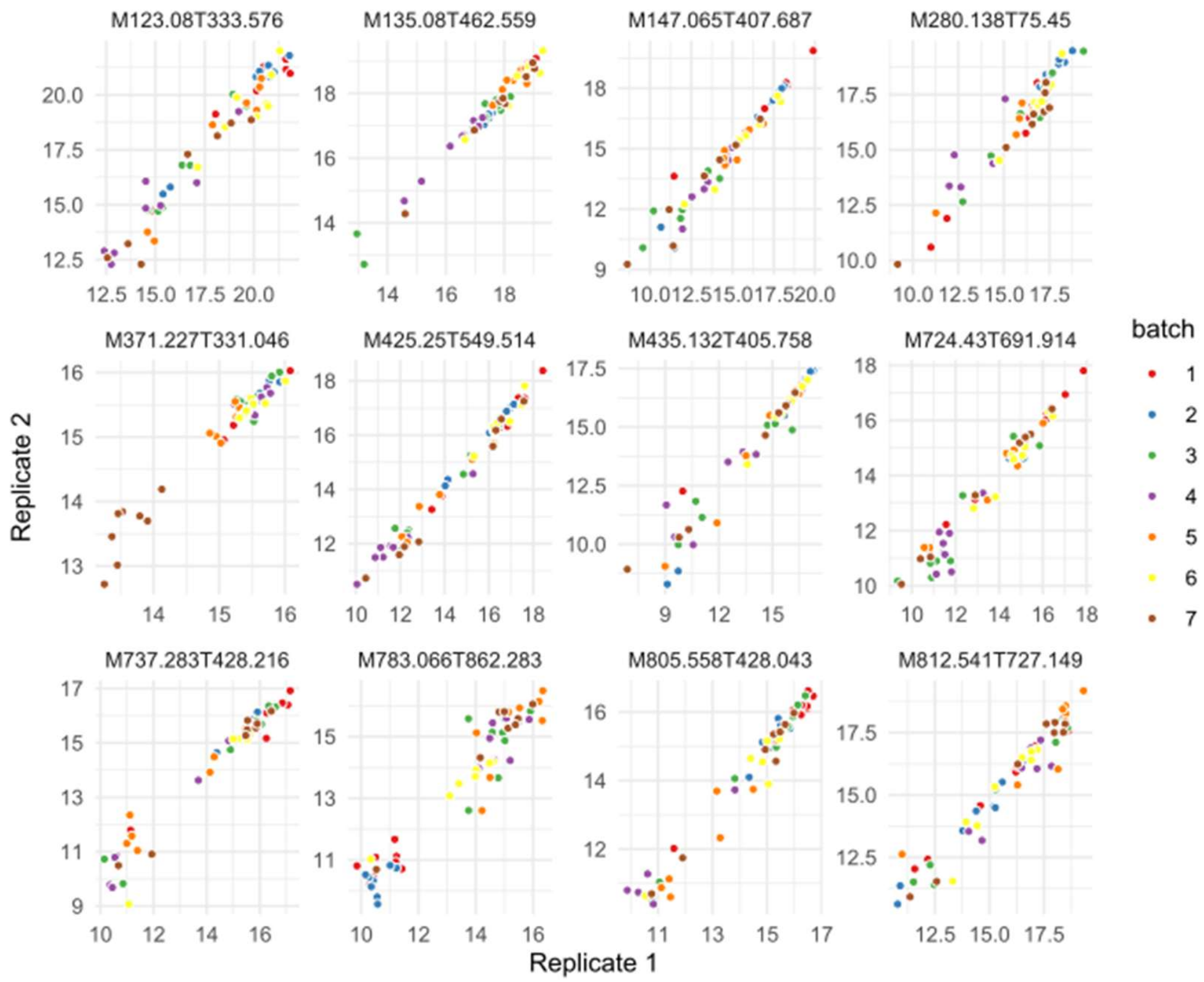

Figure S12 | Replicates of the same sample plotted against each other per feature. Each point represents material from one sample tube injected twice during a run (not in direct succession). A true raw signal that XCMS integrates consistently yields a high correlation; if either fails, the correlation is poor, reducing certainty for downstream statistical analysis. This is why we apply relatively stringent cutoffs, shown in Figure S2.

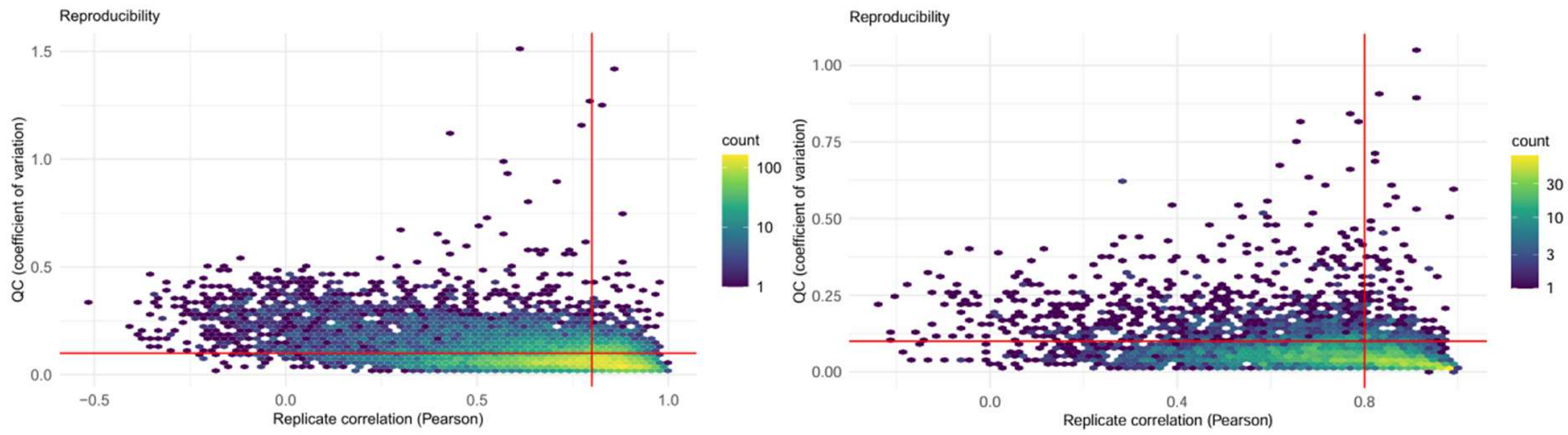

Figure S13 | Distribution of QC RSD vs. technical replicate correlation. Pos ESI in the left panel, Neg ESI in the right panel. Included features have replicate correlation > 0.8 and QC RSD < 10%.

Table S1: Additional performance metrics of lasso models

model	ROC AUC	Accuracy	Kappa	Accuracy P-Value	Sensitivity	Specificity	Precision	Recall	F1
simple_neg	0.626	0.591	0.179	0.007	0.669	0.509	0.585	0.669	0.625
simple_pos	0.565	0.569	0.135	0.038	0.653	0.482	0.566	0.653	0.606
slope_neg	0.763	0.667	0.333	0.002	0.667	0.667	0.667	0.667	0.667
slope_pos	0.667	0.615	0.231	0.027	0.641	0.590	0.610	0.641	0.625
fda_neg	0.696	0.628	0.256	0.015	0.692	0.564	0.614	0.692	0.651
fda_pos	0.692	0.628	0.256	0.015	0.590	0.667	0.639	0.590	0.613

Table S2: Cohort statistics. All five Danish regions are represented.

	Number of participants			Number of samples		
	Cases	Controls	Total	Cases	Controls	Total
N	99	99	198	271	269**	540
Gender						
F	83	83	166	227	225	452
M	16	16	32	44	44	88
Year of birth						
[1945,1955)	25	25	50	61	61	122
[1955,1965)	55	55	110	173	171	344
[1965,1975)	14	14	28	23	23	46
[1975,1995)	5	5	10	14	14	28
Donation region*						
Region H	25	25	50	85	83	168
Region M	34	34	68	64	64	128
Region N	24	24	48	75	75	150
Region S	9	9	18	27	27	54
Region SJ	7	7	14	20	20	40
Number of samples per participant						
1	60	60	120	60	60	120
5	23	23	46	115	115	230
6	16	16**	32	96	92**	188

*When counting unique participants, the most recent region of donation is considered

**Four controls missed one sample because of missingness in the storage or obstructed sample preparation.

Table S3: Mean time from sample to diagnosis in longitudinal participants

Longitudinal sample order of participants	Mean time until diagnosis (days)	Sample size
1	113.5	39
2	409.3	39
3	730.7	39
4	1034.4	39
5	1357.8	39
6	1694.3	16

Table S4: Exclusion criteria for participants, cases, and controls.

Variable	ICD-10	ATC
Alcoholism	F10	N07BB01
Psychiatric eating disorders (anorexia nervosa, Bulimia nervosa)	F50	
Anorexia	R63.0	
Testicular dysfunction	E29<br>E34.5	G03B
Ovarian dysfunction	E28	G03FA<br>G03FB
